# Spatial co-occurrence patterns of benthic microbial assemblage in response to trace metals in the Atacama Desert Coastline

**DOI:** 10.3389/fmicb.2022.1020491

**Published:** 2023-01-16

**Authors:** Ana Zárate, Verónica Molina, Jorge Valdés, Gonzalo Icaza, Sue Ellen Vega, Alexis Castillo, Juan A. Ugalde, Cristina Dorador

**Affiliations:** ^1^Doctorado en Ciencias Aplicadas mención Sistemas Marinos Costeros, Universidad de Antofagasta, Antofagasta, Chile; ^2^Laboratorio de Complejidad Microbiana y Ecología Funcional, Instituto Antofagasta and Centro de Bioingeniería y Biotecnología (CeBiB), Universidad de Antofagasta, Antofagasta, Chile; ^3^Laboratorio de Biotecnología en Ambientes Extremos, Centro de Excelencia en Medicina Traslacional, Universidad de la Frontera, Temuco, Chile; ^4^Departamento de Ciencias y Geografía, Facultad de Ciencias Naturales y Exactas y HUB Ambiental UPLA, Universidad de Playa Ancha, Valparaíso, Chile; ^5^Centro de Investigación Oceanográfica COPAS COASTAL, Universidad de Concepción, Concepción, Chile; ^6^Laboratorio de Sedimentología y Paleoambientes, Facultad de Ciencias del Mar y de Recursos Biológicos, Instituto de Ciencias Naturales A. von Humboldt, Universidad de Antofagasta, Antofagasta, Chile; ^7^Independent Researcher, Talca, Chile; ^8^Centro de Investigación y Estudios Avanzados del Maule, Vicerrectoría de Investigación de Investigación y Posgrado, Universidad Católica del Maule, Campus San Miguel, Talca, Chile; ^9^J’EAI CHARISMA (IRD-France, UMNG-Colombia, UA-Chile, UCM-Chile, UCH-Chile, IGP-Peru, UPCH-Peru) and Nucleo Milenio UPWELL, Concepción, Chile; ^10^Center for Bioinformatics and Integrative Biology, Facultad de Ciencias de la Vida, Universidad Andrés Bello, Santiago, Chile; ^11^Departamento de Biotecnología, Facultad de Ciencias del Mar y Recursos Biológicos, Universidad de Antofagasta, Antofagasta, Chile

**Keywords:** sediments microbial gradient, hyperarid coast, trace metals, microbial networks, predicted functions, biomonitoring

## Abstract

Taxonomic and functional microbial communities may respond differently to anthropogenic coastal impacts, but ecological quality monitoring assessments using environmental DNA and RNA (eDNA/eRNA) in response to pollution are poorly understood. In the present study, we investigated the utility of the co-occurrence network approach’s to comprehensively explore both structure and potential functions of benthic marine microbial communities and their responses to Cu and Fe fractioning from two sediment deposition coastal zones of northern Chile *via* 16S rRNA gene metabarcoding. The results revealed substantial differences in the microbial communities, with the predominance of two distinct module hubs based on study zone. This indicates that habitat influences microbial co-occurrence networks. Indeed, the discriminant analysis allowed us to identify keystone taxa with significant differences in eDNA and eRNA comparison between sampled zones, revealing that *Beggiatoaceae*, *Carnobacteriaceae*, and *Nitrosococcaceae* were the primary representatives from Off Loa, whereas *Enterobacteriaceae*, *Corynebacteriaceae*, *Latescibacteraceae*, and *Clostridiaceae* were the families responsible for the observed changes in Mejillones Bay. The quantitative evidence from the multivariate analyses supports that the benthic microbial assemblages’ features were linked to specific environments associated with Cu and Fe fractions, mainly in the Bay. Furthermore, the predicted functional microbial structure suggested that transporters and DNA repair allow the communities to respond to metals and endure the interacting variable environmental factors like dissolved oxygen, temperature, and salinity. Moreover, some active taxa recovered are associated with anthropogenic impact, potentially harboring antibiotic resistance and other threats in the coastal zone. Overall, the method of scoping eRNA in parallel with eDNA applied here has the capacity to significantly enhance the spatial and functional understanding of real-time microbial assemblages and, in turn, would have the potential to increase the acuity of biomonitoring programs key to responding to immediate management needs for the marine environment.

## 1. Introduction

High abundance, diversity, and activity of microorganisms in marine sediments play vital roles in global biogeochemical cycles (Prokopenko et al., 2013; Hoshino et al., 2020), partially because they are involved in the transforming and degrading organic matter (Koho et al., 2013), these processes, release nitrogen and phosphorus nutrients into the water column, thus facilitating the growth of primary producers and contributing to climate regulation (Cavicchioli et al., 2019). However, most previous studies that consider the composition and spatial distribution of microbial communities in coastal areas both worldwide (Jayakumar et al., 2012; Glass et al., 2015) and in Chile have focused on the water column (e.g., Molina et al., 2010, 2020, 2021; Stewart et al., 2012; Ulloa et al., 2012) and little is known about benthic microbial structure, activity and interactions in marine sediments that experience fluctuating redox conditions due to the oxygen-depleted waters and trace metal (TM) gradients (Farías et al., 2004; Broman et al., 2017; Rasigraf et al., 2017; Miranda et al., 2020).

In general, these conditions are generated by vertical geochemical zonation that results from the thermodynamically driven succession of organic matter (OM) availability and high-potential electron acceptors (e.g., oxygen and nitrate; Prokopenko et al., 2013; Broman et al., 2017; Rasigraf et al., 2017). Therefore, marine sediments are considered both a sink and a source for OM as well as other nutrients and pollutants, contributing substantially to climate regulation processes *via* carbon burial (Koho et al., 2013; Sander et al., 2015). In fact, previous research carried out in northern Chilean coastal sediments, indicates that OM accumulation governs the biogeochemistry of the sediments bio-essential TMs such as Cu and Fe closely associated with nutrient-rich but oxygen-depleted waters (Valdés et al., 2005, 2014; Plass et al., 2020) *via* atmospheric deposition from the Atacama Desert and continental inputs, primarily the Loa River (Romero et al., 2003; Flores-Aqueveque et al., 2014). Moreover, although the TMs are important redox-active elements, they persist and accumulate, influencing the selection, distribution and dynamics of particular microbial guilds associated with metalloenzyme-involved metabolisms, such as nitrification or denitrification besides other processes related with sulfur cycling (Glass et al., 2015; Rasigraf et al., 2020), and therefore, establishing specific metabolic networks and geochemical profiles in marine zones associated with oxygen-depleted waters (Glass et al., 2015; Rasigraf et al., 2017).

Hence, the relevant question is whether microbial benthic communities and their control mechanisms will be varied in the marine sediments with different depositional histories and fluctuating redox conditions due to the influence of oxygen-depleted waters and TMs gradients off northern Chile. We hypothesized that the fractionation of Cu and Fe acts as a driving forces in modulating the structure of benthic microbial assemblages and could be directly linked to the responses of active and resident communities. To test the hypothesis, we combined the analysis of DNA- and RNA-derived microbial 16S rRNA gene sequences from sediments of two contrasting coastal zones in northern Chile, one from the semi-enclosed Mejillones Bay, and the other that we referred to as the Off-Loa zone, as it has river input from the hyperarid Atacama Desert. We investigated the spatial microbial community characteristics, assembly patterns, gene prediction and environmental driving factors focusing on changes in response to Cu and Fe within 10–60 m water-depth gradient. Furthermore, we performed various network and statistical analyses in order to identify the significance of co-occurrence of microbial communities between contrasting study zones.

Correlation-based network analysis provides a good way to determine the potential interplays between microbes as well as their structuring and mediating ecological functions in various environments (Chaffron et al., 2010; Banerjee et al., 2018; Felipe-Lucia et al., 2020), including the Mejillones Bay and Loa River (Zárate et al., 2020, 2021). Therefore, the results of the present study highlights important targets to better understand microbial responses to the bioavailability of TMs in low-oxygen sedimentary environments. This knowledge is indispensable for understanding and predicting how ecosystems will respond to many of today’s biggest challenges, from climate change (hypoxia and warming) to the propagation of metal and antibiotic resistance mechanisms between microbes (Nogales et al., 2011; Hernando-Amado et al., 2019).

## 2. Materials and methods

### 2.1. Study area

Fieldwork was conducted in two upwelling coastal sites off northern Chile (Supplementary Figure S1) in late autumn 2017: named Mejillones Bay (MB; 23°20′ S, 70°30′ W) and Off Loa Zone (OL; 21°20′ S, 71°05′ W). These sites were chosen as they fall located in a natural laboratory area.

MB is located in the southeastern Pacific on the coast of the Atacama Desert and has been considered a semi-enclosed system with high biological production (Marín et al., 2003), strongly anthropized *via* the development of multiple mineral exporting harbors and a thermoelectric plant, leading to significant implications on the heavy metal (HM) accumulation in sediments (Valdés, 2012; Castillo et al., 2019; Zárate et al., 2021). OL is located approximately 210 km north of MB near the city of Iquique (Isla et al., 2010). This area, though less studied than MB, has been described as a spawning and nursery area for small pelagic fish with high planktonic production (Santander et al., 2017), supporting world’s largest fisheries (Herrera and Escribano, 2006; Palma et al., 2006). In addition, is exposed to the discharge of the Loa River, which extends east to west through the Atacama Desert and to the Pacific Ocean, carrying different pollutants originating from industrial mining (e.g., the Chuquicamata copper mine), agriculture and domestic wastes. However, due to the water usage the Loa River discharge to the coastal zone is mainly sporadic owing to precipitation increases and flood (up to four orders of magnitude) mainly during austral summer (i.e., December and March; Romero et al., 2003).

#### 2.1.1. Sample collection

Three sediment cores 1–5 cm long and with 2.5 cm inner diameters were collected at each field site using an Ekman Box-core (Bottom sampler Ekman-Birge, Hydro-Bios) at depths of 20 and 60 m in the middle of bay (MB), and at 20, 30, and 60 m depth Off OL between July and November 2017, respectively. The sediment for DNA and RNA were immediately preserved at 4°C during transport to the laboratory. Subsequently, the uppermost 3 cm of the sediment cores were cut into slices every 1 cm and stored at −20°C for DNA and −80°C for RNA, the latter preserved with RNAlater® solution (Ambion, United States) until the extraction process. Sediment subsamples from each of the same core fraction used in the nucleic acid analyses were used for measuring Cu and Fe concentration, total carbon (TC), nitrogen (TN), Sulfur content (TS) and grain size distribution. Briefly, the content of TC, TN, and TS was measured by analyzing ~3 mg of dry sediment on a LECO CHNS elemental analyzer (2400 series II, Perkin Elmer). Then the analytical procedure was verified with the analysis of sulfanilic acid (C_6_H_7_NO_3_S) as a reference material (Castillo et al., 2019). At the collection sites, pH, salinity, conductivity, dissolved oxygen (DO), and temperature (°C) of the water column were measured using a CTD-O profiler (SBE 19 plus V2 Sea cat, SeaBird).

### 2.2. eDNA/RNA extraction and sequence-data processing

**Figure 1:**
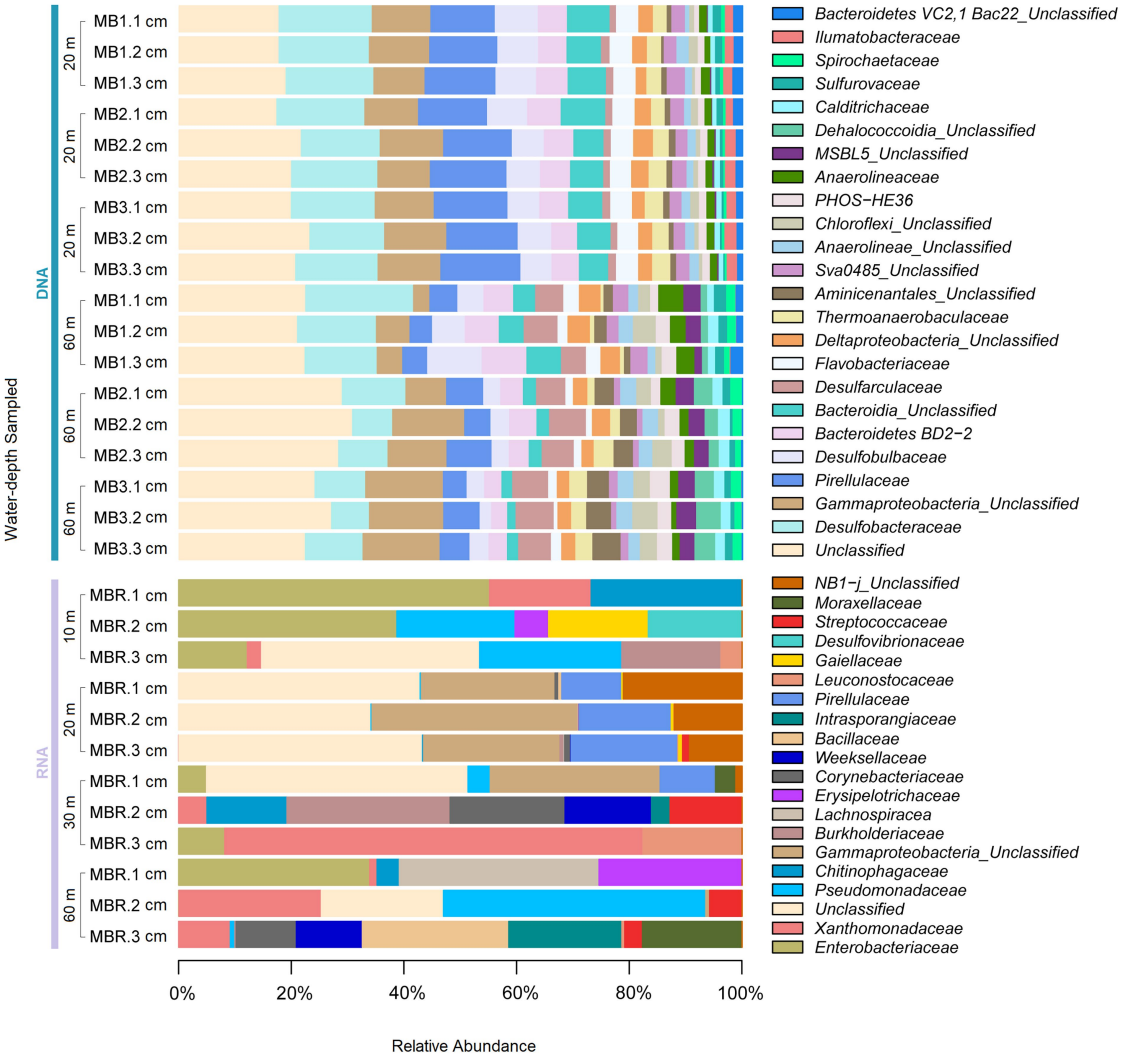
The specific composition of the benthic microbial community at family level in DNA and RNA samples across the water depths sampled in Mejillones Bay.

**Figure 2:**
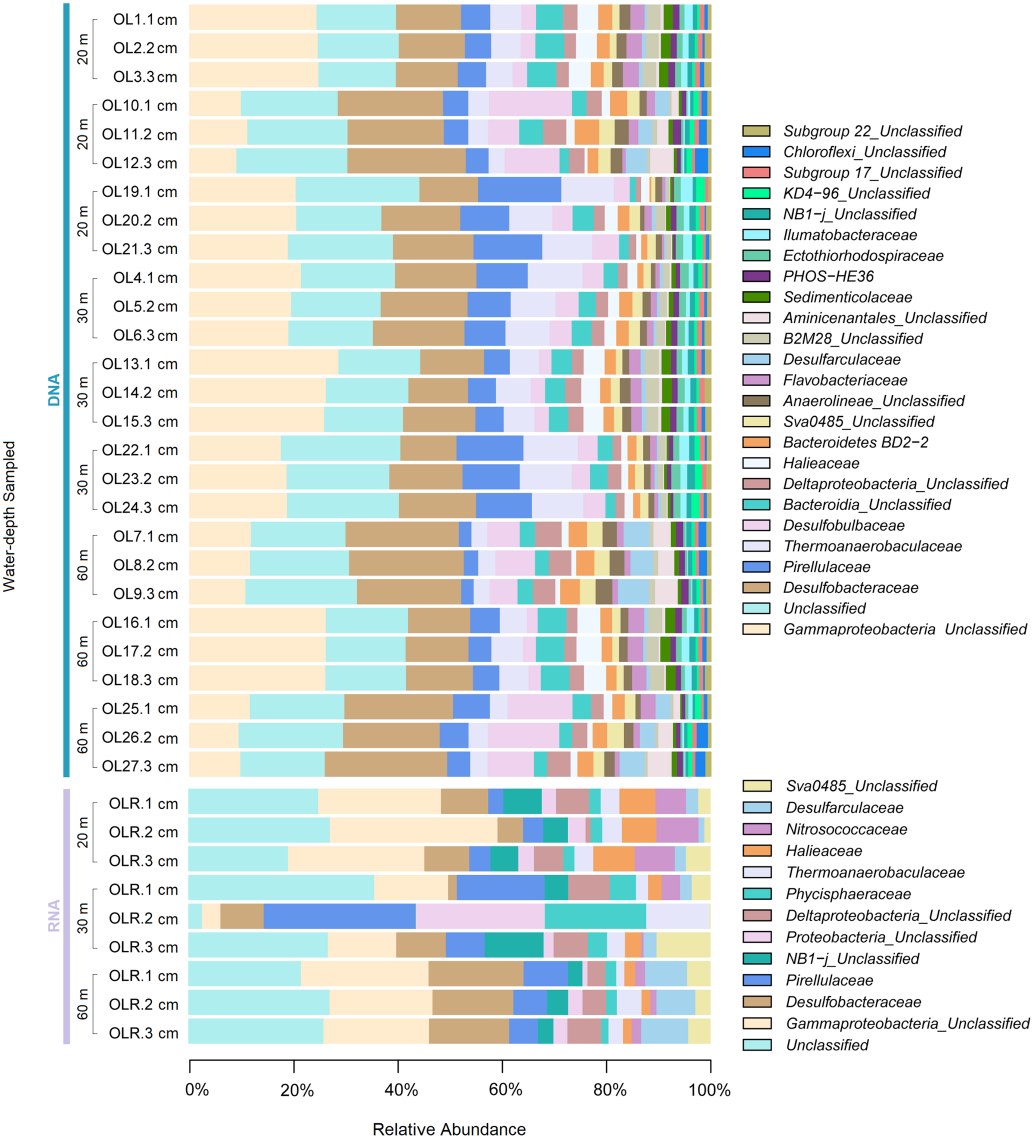
The specific composition of the benthic microbial community at the family level in DNA and RNA samples across the water depths sampled in Off-Loa River zone.

Total environmental DNA and RNA (eDNA/eRNA) was extracted in triplicate from approximately 0.5 g (wet weight) of the samples at each depth (i.e., 20, 30, and 60 m) for a total of 66 samples from two sites (see Figures 1, 2) across the Atacama Desert Coastline, employing the MoBio PowerSoil® DNA Isolation Kit (MO BIO Laboratories, Inc.) according to the manufacturer’s instructions. We supplemented with three 5-min rounds of bead beating. The yield and quality of the extracted DNA samples were reviewed on 0.8% agarose gel. The extracted genomic DNA was quantified and checked for purity at A260/280 nm by a NanoDrop spectrophotometer (Thermo Fisher 150 Scientific, United States). The DNA was stored at −20°C until further sequence processing.

eRNA samples were thawed on ice, and the remaining RNAlater® was carefully removed and discarded after initiating the RNA extraction from 2 g of sediment (wet weight) using mirVana™ miRNA Isolation Kit (Ambion, United States) following the manufacturer’s specifications with the addition of a step of mechanical cell disruptions (200-μm-diameter zirconium beads, Low Binding Zirconium Beads, OPS Diagnostics) for three rounds of 30 s (∼3,000 rpm) using a Mini-Beadbeater-8 (Biospec Products). DNA traces were removed using the Turbo DNA-free kit (Ambion, United States) and RNA concentrations were verified using the Qubit® RNA Assay Kit (Thermo Fisher Scientific, United States). Quality was ensured *via* agarose gel electrophoresis, following manufacturer’s instructions and immediately reverse transcribed to complementary DNA (cDNA) using 20 ng of total RNA with random primers provided by the ImProm-II™ Reverse Transcription System (Promega Corp, United States). Synthesized cDNA was further used for amplification according to the protocol.

The DNA samples were sequenced using next-generation Illumina MiSeq platform at Macrogen Corporation (South Korea). Sequencing of amplicons encompassing the V3–V4 hypervariable regions of 16S rRNA gene (~460 bp) of the prokaryotes were amplified using the 515 F/806 R primer pair (Caporaso et al., 2011). Meanwhile, cDNA sample analyses were performed using an Illumina MiSeq platform (pair ends 2 × 126 bp) technology MrDNA (Shallowater, Texas, United States). Illumina-generated rRNA-amplicon sequences (iTags) were further processed through bioinformatics analysis.

### 2.3. Microbial community sequence and biostatistics analyses

Sequencing analyses to assess the structure and composition of the benthic microbial communities from two field sampling coastal zones (MB and OL). Raw sequences were demultiplexed, joined paired reads, quality-filtered, chimera checked, curated and analyzed using the DADA2 pipeline (Callahan et al., 2016).

All sequences were filtered to truncate the paired reads to 150 nt and eliminate reads with quality values of <2. Error rates were estimated and corrected by pooling all the reads from the sequencing run, with default parameters. Taxonomy was assigned using the SILVA release v132 references alignment (Quast et al., 2013). Resulting amplicon sequence variants (ASVs) were analyzed using the Phyloseq package in R (McMurdie and Holmes, 2013). Noteworthy, taxonomic data were analyzed by both order and family, levels that were chosen based on the number of classified ASVs (Supplementary Table S1). For ASVs that were not assigned taxonomies of these categories, we used the last level classification (ending in “_Unclassified”).

Within-sample diversity (alpha diversity) was determined by calculating the Shannon and Simpson diversity indices (evenness) using the total number of observed ASV and Chao1 (richness; Shannon and Weaver, 1949; Oksanen et al., 2020) in the vegan R packages (R Core Team, 2015). Beta-diversities of communities were compared using the non-Metric Multidimensional Scaling (nMDS) to visualize the levels of dissimilarity of communities with Bray-Curtis distance between samples. Analysis of similarity (ANOSIM) was used to test the significance of differences between groups of sampling zones (MB and OL) of the NMDS analyses. Venn diagrams were also constructed using the InteractiVenn (Heberle et al., 2015) based on the distribution of families showing specific and shared ASVs between eDNA/eRNA and sampling zones.

Benthic microbial communities and their correlation with environmental variables were explored by Spearman’s correlation and permutational multivariate analysis of variance using distance matrices (PERMANOVA) to test the significance of variables using R statistical software. In addition, redundancy analysis (RDA) was performed for a better understanding of the correlation among abundant bacterial families and physicochemical parameters in different sampling zones using CANOCO v4.5 for Windows *via* Monte Carlo permutation testing with 999 permutations (Ter Braak and Smilauer, 2002). Variables with a variance inflation factor (VIF) of >20 were removed from the dataset one at a time in order to control for correlated explanatory variables which could inflate the variance of the canonical coefficients.

To highlight the substantial shifts in benthic microbial community assemblage, linear discriminant analysis (LDA) effect size (LEfSe; Segata et al., 2011) was used to identify taxa with significant differences between DNA and RNA at various taxonomic levels. In addition, a differential abundance analysis was performed with the DESeq2 (Love et al., 2014) of the R library to identify the ASVs at the family level that have significant fold changes between the sampling zones (MB vs. OL) and water-depths. The log2 fold change plot was made with the significant ASVs at a value of *p* < 0.01. Finally, microbial community functional potential profiles were predicted based on 16S rRNA gene data using PICRUSt and the functional terms stored in the Kyoto Encyclopedia of Genes and Genomes (KEGG) database (Langille et al., 2013). The heatmap was created based on Bray–Curtis dissimilarity dendrogram of the functional capabilities detected.

A co-occurrence network was applied to reveal the interaction between microbial communities. The network inference was estimated based on co-occurrence patterns based on Spearman’s significant correlations (R > 0.75; *p* ≤ 0.001) at the family level using Fruchtermann–Reingold layout (Fruchterman and Reingold, 1991). In order to describe the topology of the resulting microbial network from the study zones (Supplementary Table S2), a set of metrics including edges, nodes, degree, betweenness centrality (BC), clustering coefficient (CC), average path length, modularity and network diameter was calculated (Newman, 2003). Nodes with highest BC value, representing the relevance of a node as capable of holding together with communicating nodes, were recognized as possible “keystone taxa” in co-occurrence networks (Brandes et al., 2001). The network visualization and modular analysis were conducted in Gephi v0.9.2 software (Bastian and Heymann, 2009).

### 2.4. Chemical procedures for sequential extraction of Fe and Cu in sediment cores

The exchangeable fraction of Fe and Cu in sediments was determined using the BCR three step sequential extraction procedure described by Rauret et al. (1999) plus the pseudo-total fraction, which was obtained after digestion with aqua regia.

Details of the experimental protocol for sequential extraction and the reagents applied in this study have been described in a previous study carried out by Pardo et al. (2004). Briefly, all extractions were drained into 50 ml metal-free polypropylene tubes (VWR), where the ground sediment samples (1 g) were mixed for 16 h (overnight) at room temperature, using a mechanical shaker. The extract was then separated from the solid residue by centrifugation at 3,000 rpm for 15 min, and each resultant supernatant liquid (each extraction step was evaporated to near dryness) was transferred into a new vial, then sealed and stored at −20°C until chemical analyses. Subsequently, the residue sediment was washed twice by adding 10 ml of deionized water (Mili-Q quality), shaken for 15 min on the end-over-end shaker and centrifuged for 20 min at 3,000 rpm. The supernatant was decanted, taking care not to lose the solid residue to use in the next step, testing visually the clear solution indicating the total digestion (no residual) of the sediment.

Three independent replicates were performed for each sample (1 g sediment) and blanks were measured in parallel for each set of analyses using aqua regia extraction and the BCR procedure, respectively. Laboratory blanks and replicates were analyzed during each batch of extractions. Finally, the residual fraction (RF) was determined separately by digestion with aqua regia for 12 h, using a CEM-MARS 5 brand microwave digestion system with closed Teflon vessels and covers used for acid digestion of the sediment samples. Vessels containing 1 g of the sediment and 12 ml of acid (15 ml HNO_3_ + 5 ml HCl) were heated at 60°C for 2 h and then at 100°C for 2 h. After cooling, digests were filtered through Whatman filter paper into 100 ml volumetric flasks.

Fe and Cu concentrations in the different extracted solutions were quantified by inductively coupled plasma optical emission spectrometry (ICP-OES), using a PerkinElmer Optima 8300DV spectrometer. All dilutions were prepared with deionized water. The plastic and glassware used were soaked in 5% HNO_3_ solution overnight and rinsed with distilled water prior to use. To evaluate the accuracy and precision of the BCR extraction in all samples, the marine sediment MESS-3 standards (National Research Council, Canada) were used following all the lab procedures. Measured concentrations in BCR-701 included in the analysis were in good agreement against their certified values, and the standard deviation of replicated samples was on average ± 7%.

## 3. Results

### 3.1. Environmental characteristics of water and trace metals in surface sediments

Water-depths ranged from 20 to 60 m. The variations of temperature (T°C) and DO (Supplementary Table S3) distinguished the two sampling zones, MB and OL. The DO levels in the overlying bottom water were found to vary from 1.11 to 0.160 mL/L in MB, while 0.078 to 0.065 mL/L was the observed range across the depth in OL. The salinity in the study locations varied slightly from 34.83 to 34.86 PSU. Meanwhile, the temperature decreased with depth in MB (15.03°C–14.07°C between 20 and 60 m), whereas it remained stable at both depths in OL with a range of 12.53–12.78°C.

TC and TS concentrations in the sediments of both sampling localities increase with depth and were much higher in MB than OL; however, only TC showed a significant difference between sampling zones (*p* = 0.003). Sediment values of TN in MB were also slightly higher than those in OL (*p* = 0.005), ranging from 3.43 to 3.82 and 0.26 to 0.46, respectively (Supplementary Table S3).

Cu and Fe were measured to establish their exchangeable fractions and assess their potential bioavailability in surface sediments of MB and OL. Analysis of marine sediment standard reference material showed satisfactory accuracy, with the recoveries for Fe and Cu being up to 100%. The average pseudo-total elemental (total extraction) concentration in the sediment of MB for Fe and Cu were observed as 4351.09–5132.34 mg kg^−1^ and, 46.79–50.54 mg kg^−1^ at 20 and 60 m depth, respectively. Meanwhile, OL sediments presented pseudo-total contents of Fe and Cu to be 7568.95–7133.01 mg kg^−1^ and 49.57–53.61 mg kg^−1^ at 20 and 60 m, respectively (Table 1). As well as most Fe and Cu content was bound within the crystal structure of the minerals as a residual fraction (RF), being 43% and 46% in MB and 57% and 58% in the OL at 20 and 60 m, respectively. Results indicated that, the fractions in the sediments of both sampling localities followed a trend of TMs magnitude concentration from RF > oxidizable > reducible > exchangeable with slight differences (see Supplementary Figure S2). The exchangeable fraction of Fe and Cu was higher at 20 m (52%) than 60 m (51%) in both sampling localities. The reducible and oxidizable fractions showed similar percentages with 60% and 75% at 20 and 60 m depths.

**Table 1:** Concentrations of Cu and Fe in each fraction of surface sediments of Mejillones Bay and Off-Loa River zone (mg.kg^−1^; *n* = 3).

Depth (m)	Fraction		Mejillones Bay		Off-Loa zone	
			Cu	Fe	Cu	Fe
20	F1	Mean, SD	5.99 ± 0.11	945.89 ± 47.31	5.13 ± 0.26	803.71 ± 56.81
		Range	5.92–6.11	911.29–999.80	4.88–5.40	744.88–858.25
	F2	Mean, SD	8.90 ± 0.63	960.51 ± 11.30	4.95 ± 0.08	939.31 ± 48.87
		Range	8.34–9.58	950.35–972.69	4.91–5.04	883.25–972.90
	F3	Mean, SD	11.69 ± 0.33	1029.54 ± 10.45	6.60 ± 0.16	1282.30 ± 61.16
		Range	11.31–11.96	1017.47–1035.73	6.45–6.76	1213.90–1331.72
	RF	Mean, SD	23.39 ± 1.97	2175.55 ± 136.25	49.57 ± 0.97	7568.95 ± 1239.01
		Range	21.14–24.56	2057.93–2324.85	48.48–50.35	6143.75–8389.98
60	F1	Mean, SD	6.21 ± 0.02	992.59 ± 16.50	5.07 ± 0.15	823.62 ± 38.14
		Range	6.18–6.22	976.34–1009.33	4.91–5.20	797.68–867.41
	F2	Mean, SD	9.85 ± 0.44	999.85 ± 33.82	5.18 ± 0.23	931.28 ± 14.58
		Range	9.41–10.29	968.39–1035.61	4.92–5.37	921.39–948.03
	F3	Mean, SD	12.56 ± 0.38	1061.87 ± 3,857	6.95 ± 0.06	1326.37 ± 22.40
		Range	12.18–12.94	1060.29–1102.20	6.87–6.99	1308.56–1351.51
	RF	Mean, SD	25.42 ± 0.18	2566.17 ± 63.16	53.61 ± 0.86	7133.01 ± 1368.18
		Range	25.29–25.63	2494.36–2613.07	52.61–54.16	6263.44–8710.07

### 3.2. Diversity and spatial patterns of benthic microbial community structure

DNA sequencing yielded 1,309,920 and 1,083,719 microbial raw reads in MB and OL, respectively. After denoising and chimera remotion, a total of 1,287,708 in MB and 951,723 in OL from 16S rRNA gene sequences were employed for downstream analyses. RNA sequencing yielded 727,041 and 391,476 microbial raw reads to sediment samples in MB and OL, respectively. After the same quality control, a total of 593,410 and 315,869 high-quality sequences remained in MB and OL, respectively (see Supplementary Table S4).

To compare the diversity and richness of benthic microbial communities in the different sampling localities across water-depth, Chao1, ACE, Simpson, and the Shannon diversity indices were calculated based on the generated ASVs (Supplementary Figure S3). The richness, estimated by the number of ACE and Chao1, were significantly different (*p* < 0.001) among the eDNA and eRNA from MB and OL, yet had no significant correlation with the sampled water-depth gradients. In general, these parameters indicate that the resident (eDNA) microbial abundance and diversity in both study zones were higher than that in active (eRNA) fraction of the communities studied.

Both benthic sampling zones harbored diverse microbial communities, which were identified from eDNA and eRNA (Figures 1, 2). The classification of 16S rRNA gene fragment sequences from MB sediment samples were assigned to 48 bacterial phyla and 6 Archaea groups and further classified into 123 classes, 264 orders, and 366 families of prokaryotes. In OL, a total 47 bacterial phyla and 6 Archaea groups were observed, which included 122 classes, 265 orders and 385 families. Microbial taxa exhibiting a relative abundance >1% in each sediment sequences at various taxonomic levels were designated as dominant. The benthic microbial communities at both sampling zones consisted mainly of the same microbial communities, yet their distributions differed (Figure 1). *Proteobacteria* accounted for 28.5% and 43.4% of the relative abundance in MB and OL, respectively. Within this phylum, the sequences mainly consisted of *Deltaproteobacteria* (18.6% and 21.7%), *Gammaproteobacteria* (9.3% and 21.1%), and *Alphaproteobacteria* (0.4% at both locations).

However, differences in the relative contribution of dominating classes were found in the different templates, for example, *Gammaproteobacteria* was higher (23.0%) in eRNA than in eDNA samples (9.3%), whereas *Deltaproteobacteria* (18.6%), *Bacteroidia* (10.0%) and *Planctomycetacia* (6.3%) were higher in eDNA compared with eRNA templates (3.5, 5.3 and 1.0%, respectively). Also, eDNA recorded higher proportions of *Deltaproteobacteria* (21.7%), *Gammaproteobacteria* (21.1%), *Bacteroidia* (6.6%), and *Planctomycetacia* (5.3%) compared to the RNA fraction (7.5, 9.8, 1.3 and 2.4%, respectively). The retrieved archaeal in the sequencing were detected at very low frequencies (always <0.5%). The archaeal groups were affiliated with *Thermoplasmata*, *Bathyarchaeia*, *Lokiarchaeia*, *Nitrososphaeria*, *Nanohaloarchaeia*, *Woesearchaeia*, *Methanomicrobia*, *Micrarchaeia*, *Odinarchaeia*, and *Heimdallarchaeia*. The rest of the groups were rare and represented by <0.04% of relative abundance at both sampling localities.

### 3.3. Differential distribution of ASVs at family level in the sampling zones across sampled water-depth

At both sites, the most abundant microbial families by far (>3% relative abundance) in eDNA samples were *Desulfobacteraceae*, *Pirellulaceae*, *Desulfobulbaceae*, and *Thermoanaerobaculaceae*. However, in eRNA samples, Enterobacteriaceae, *Xanthomonadaceae*, *Pirellulaceae*, and *Pseudomonadaceae* were the most abundantly active families (Figures 1, 2). Additionally, although many validated reads were classified at the phylum level, the number of reads affiliated with unclassified taxa increased from the phylum to the genus level (see Supplementary Figure S4), recording 25.7 and 16.7 from DNA and RNA, respectively, at the class level.

**Figure 3:**
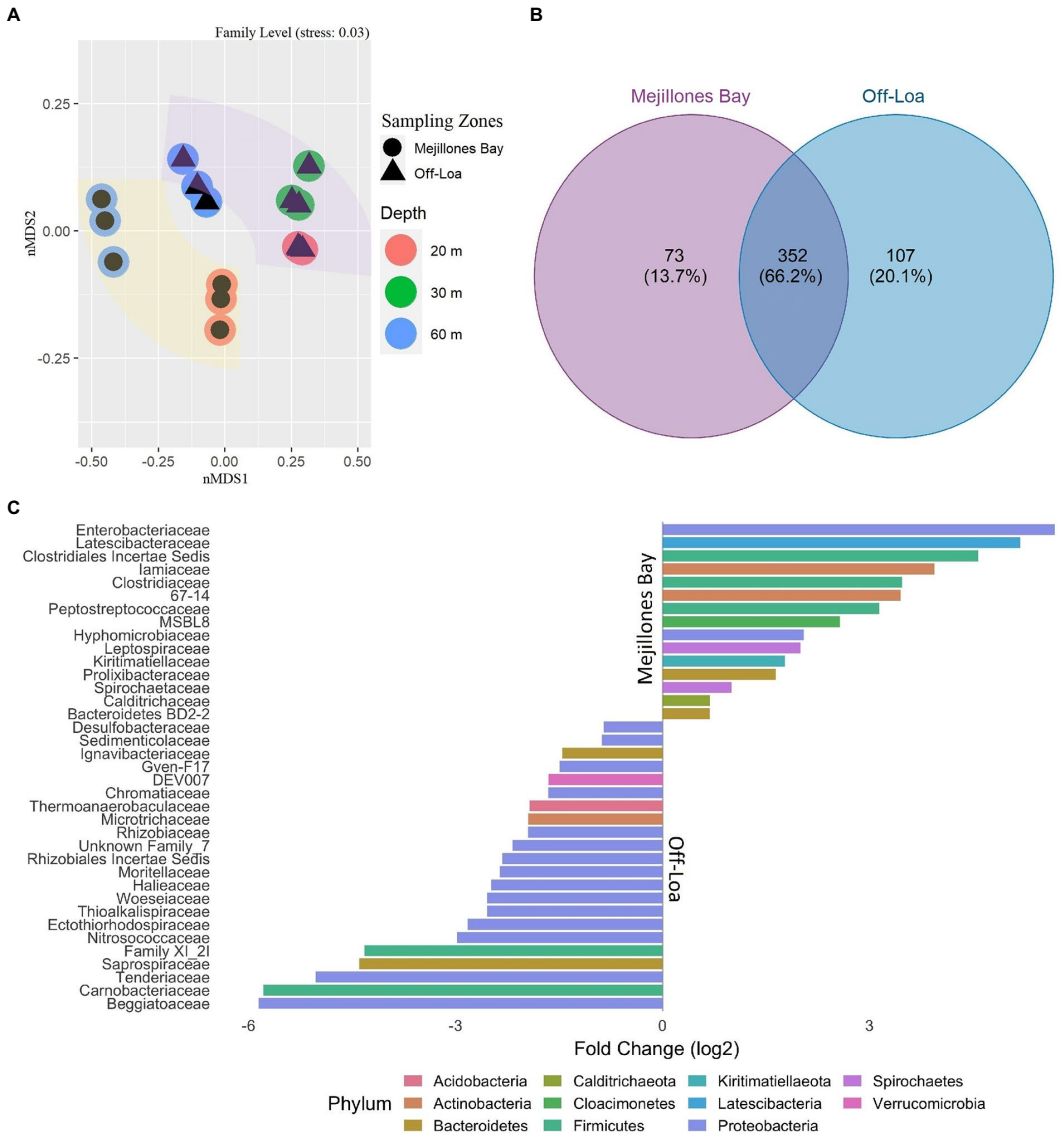
Microbial assemblage structure in Mejillones Bay and Off-Loa River zone. **(A)** Non-metric multidimensional scaling (nMDS) analysis of different microbial communities at different water-depths. **(B)** Venn diagram showing the degree of overlap of ASVs at the family level in the study zones. **(C)** DESeq2 analysis showing the family differential abundance based on sampling zones (positive fold change values more abundant in MB and negative fold change values more abundant in OL).

**Figure 4:**
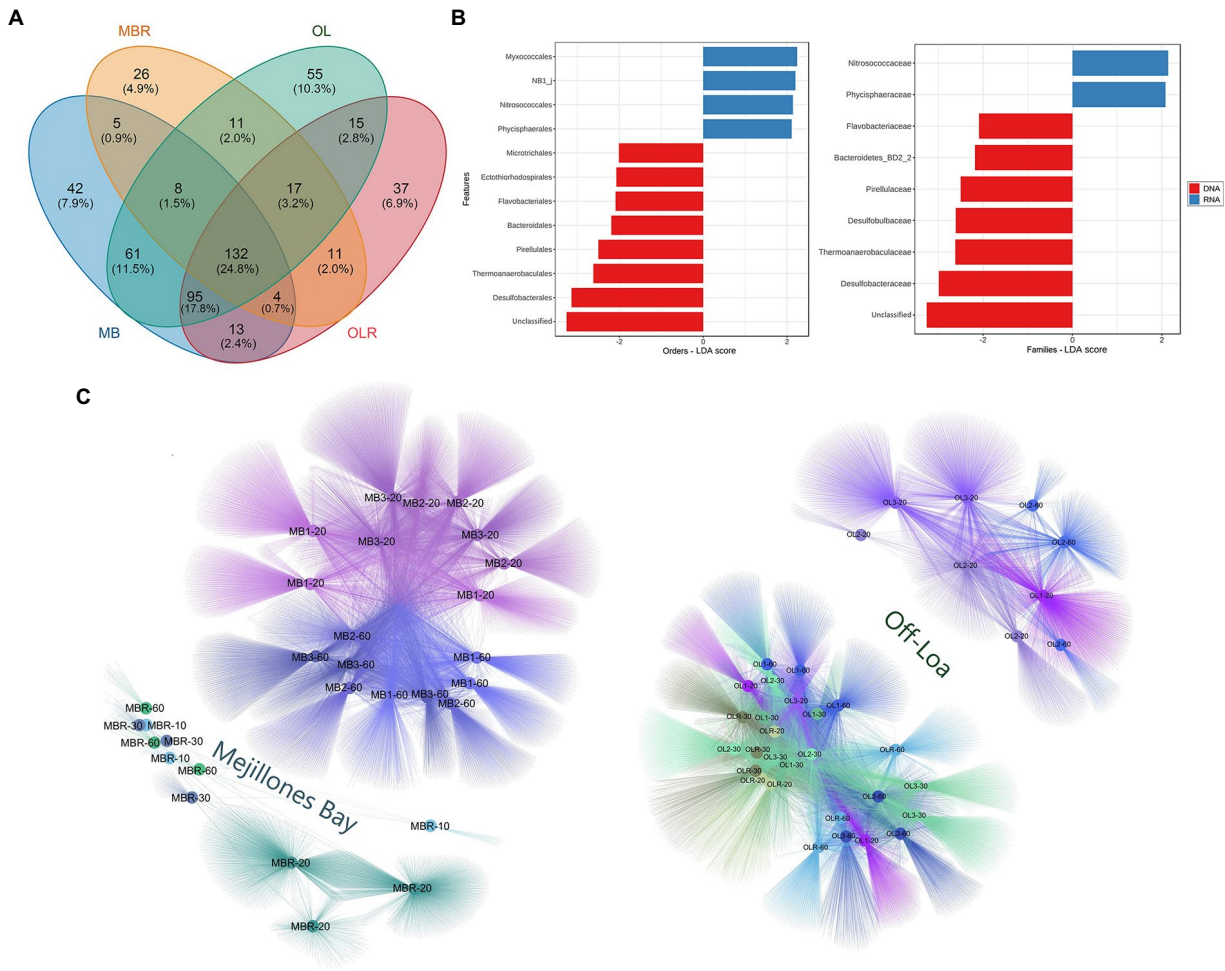
Microbial assemblage structure in Mejillones Bay and Off-Loa River zone. **(A)** Venn diagram with degree of overlap of families in eDNA (MB-OL) and eRNA samples (MBR-OLR). **(B)** Cladogram of linear discriminant analysis (LDA) score showing the significantly different orders and families in the DNA (red) and RNA (blue). **(C)** Microbial patterns of presence/absence of communities in eDNA and eRNA from the study zones.

Dissimilarity analysis based in nMDS plots through the ANOSIM test using study areas and depth categories are shown in Figure 3A. These analyses indicate that the microbial community in the eDNA fractions from sediment samples was significantly different (*p* < 0.001) from the eRNA fractions with a clear separation of the assemblages with the depth (Supplementary Figure S5). Moreover, the Venn diagram (Figures 3B, 4A) showed that 302 ASVs at level family (66.2%) were shared among the two sampling zones, 73 and 107 ASVs were exclusively recorded in MB and OL, representing 13.7% and 20.1% of the microbial community.

Overall, the communities from the two sampling localities presented a 32.56% dissimilarity when compared to each other. DESeq2 analysis showed that the six major families responsible for the significant spatial variations in MB were *Enterobacteriaceae*, *Latescibacteraceae*, *Clostridiales Incertae Sedis*, *Iamiaceae*, *Clostridiaceae*, and *Solirubrobacterales_67–14*. *Beggiatoaceae*, *Carnobacteriaceae*, *Tenderiaceae*, *Saprospiraceae*, *Family XI_2I*, and *Nitrosococcaceae* were the corresponding representatives in OL (Figure 3C). Indeed, *Nitrosococcaceae* and *Phycisphaeraceae* were the families differentially active in RNA compared with DNA samples (Figure 4B). Such differences were also noticeable in the presence and absence network of microbial assemblage both eDNA and eRNA (Figure 4C).

Across the depths sampled, major shifts in the relative abundance of families were also observed; At 20 m in MB, the major differences consisted of *Microtrichaceae*, *Unknown Family_7* (*Gammaproteobacteria*), *DEV007*, *Rhizobiaceae* and *Nitrosococcaceae*. In OL at the same depth, however, *Saprospiraceae*, *Eel-36e1D6n* (*Myxococcales*), *Desulfomicrobiaceae*, *Tenderiaceae*, and *Marinifilaceae* were differentially abundant (Supplementary Figure S6A). The families that characterize the differences at 60 m consisted of *VHS-B3-70* (*Myxococcales*), *Latescibacteraceae*, *Omnitrophaceae*, *Desulfuromonadaceae* and *Thermoflexaceae* in MB. In OL at 60 m, *AB-539-J10* (*Dehalococcoidia*), *Beggiatoaceae*, *TG3* (*Fibrobacterales*), *Carnobacteriaceae*, *Bacillaceae* and *UBA12409* (*Babeliales*) were responsible for the differences observed (Supplementary Figure S6B).

### 3.4. Linking microbial assemblages to metal fractioning and physico-chemical factors

**Figure 5:**
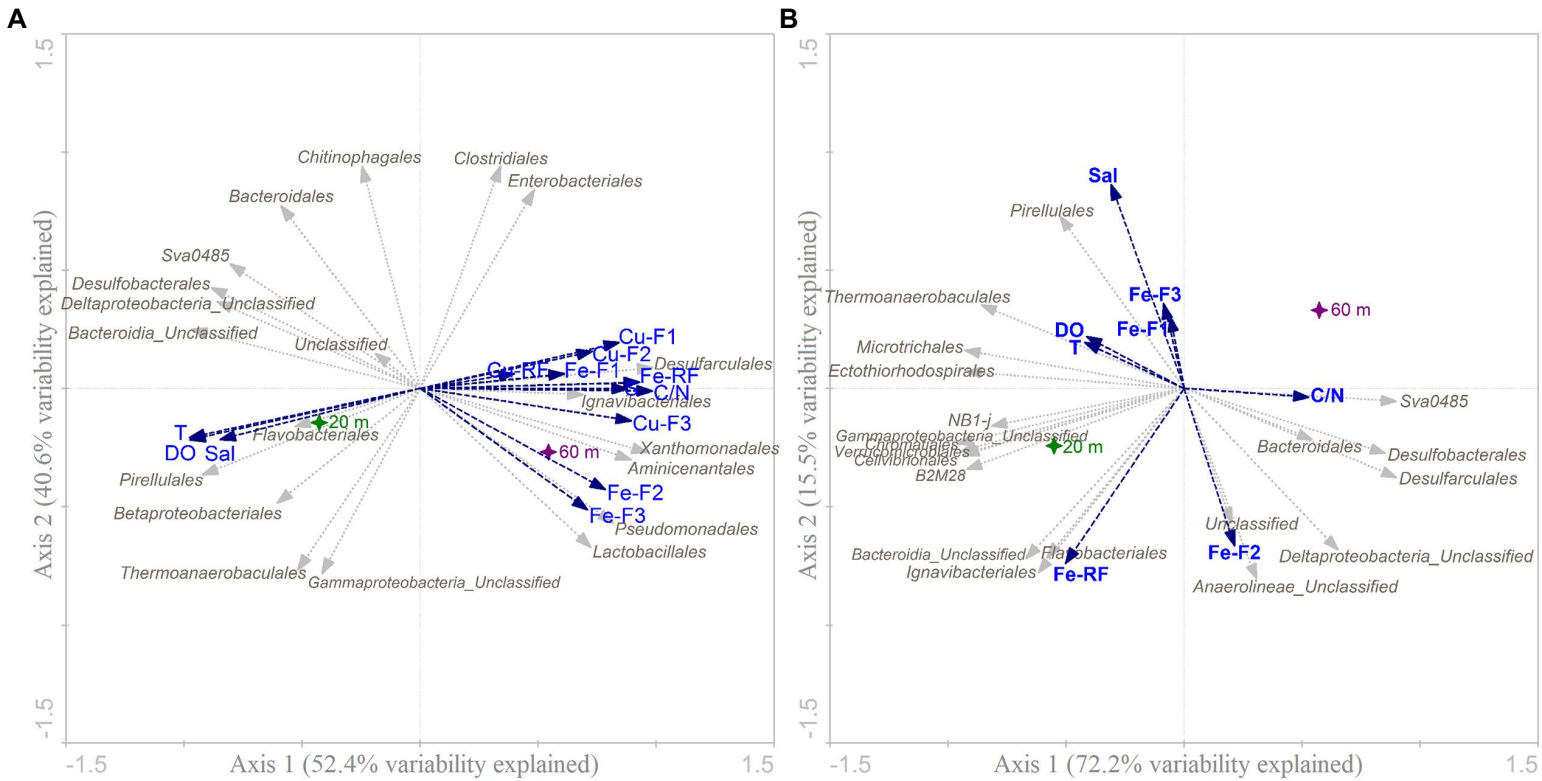
RDA ordination plot for the first two principal dimensions of the most significant physicochemical variables shaping benthic microbial assemblage composition and structure in the sediments of **(A)** Mejillones Bay and **(B)** Off-Loa River zone across water depth sampled. The symbols indicate the sampling sites, and the arrows indicate the environmental variables and their relative effects on the community.

RDA analysis of the benthic microbial community structure variability in the study area is shown in Figure 5. The first two axes of the RDA account for 93.0 and 87.7% of the total variation in MB and OL, respectively, with the RDA axis 1 accounting for 52.4% and 72.2% of the variation of each. The overlying environmental variables associated with TM and other physicochemical parameters indicates the relationship of factors with the greater impact in the benthic microbial community structuring variability.

In MB (Figure 5A), all TM fractions (F1, F2, F3, and RF) combined with the C/N ratio showed a positive relationship with the microbial assemblage at 60 m depth, mainly composed by members of the *Desulfarculales*, *Ignavibacteriales*, *Xanthomonadales*, *Aminicenantales*, *Pseudomonadales*, and *Lactobacillales*. In addition, temperature, DO, and salinity correlated negatively with *Flavobacteriales*, *Pirellulales*, *Betaproteobacteriales*, and *Thermoanaerobaculales* orders at 20 m.

Conversely, a different result was observed in OL where the metal fractioning was not clearly associated with the microbial communities across the depth sampled (Figure 5B). The ASVs were grouped with the shallow zone sampled (20 m). Salinity, DO, temperature, and the F1, F3 and RF of Fe fraction were associated with *Flavobacteriales*, *Pirellulales*, *Thermoanaerobaculales*, *Microtrichales*, *Ectothiorhodospirales*, and *Ignavibacteriales*, whereas Fe-F2 and C/N accounted for the distribution of *Sva0485*, *Desulfarculales*, *Desulfobacterales*, *Anaerolineae*, and *Deltaproteobacteria* Unclassified.

The Mantel test analysis, recorded no significant correlation between microbial structure and the measured environmental variables. However, a combination with PERMANOVA testing of significance highlighted that Cu-F1 (*p* = 0.021*), Cu-F2 (*p* = 0.01**), Cu-F3 (*p* = 0.005**), Fe-F3 (*p* = 0.037*), C/N ratio (*p* = 0.011*), temperature (*p* = 0.027*), TC (*p* = 0.003**), and TN (*p* = 0.005**), explained a high proportion of microbial community variation in both sampling locations. The relative importance of each physicochemical parameter and bioavailable metal(loid) to diversity indices (Chao1, Observed and Shannon) was also tested using PERMANOVA. Furthermore, Spearman’s rank-order correlations were determined between environmental variables and microbial communities at both locations, which then corroborated that microbial communities were significantly associated both positively and negatively with salinity, temperature, DO, TN, TS, TC, with Cu and Fe fractioning in their distinct variations (Supplementary Figure S7).

### 3.5. Co-occurrence networks and predicted metabolic potential of benthic microbial communities

**Figure 6:**
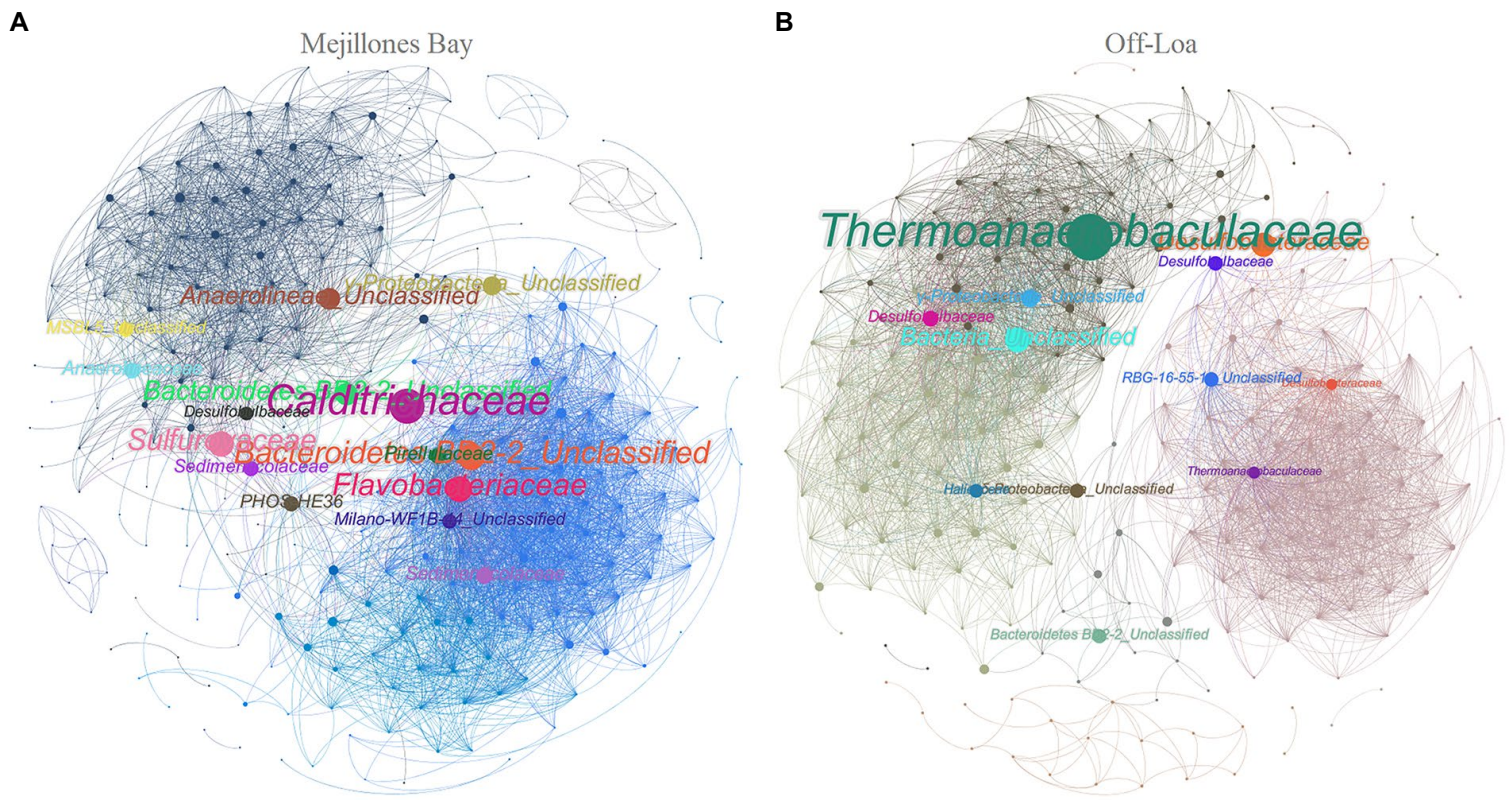
Sedimentary network of the microbial co-occurrence patterns in **(A)** Mejillones Bay and **(B)** Off-Loa River zone. Nodes (bubbles) represent ASVs at the family level and edges (lines) represent the strong (Spearman’s *p* > 0.75) and statistically significant (FDR adjusted *p* < 0.01) correlations. Module hubs were colored according to modularity class and the size of each node is proportional to the value of BCC.

Network constructed based on the co-occurrence patterns of microbes in surface sediments from MB and OL are shown in Figure 6. The analysis revealed that both study zones had unique co-occurrence patterns, composed by two large central module hubs. Nevertheless, OL had the highest values of modularity (i.e., higher complexity) and degree centrality (0.521 and 15.91, respectively) compared to the MB (0.461 and 15.21, respectively). Moreover, the average clustering coefficient, which was used to describe how well nodes related to their neighbors, indicated marginally lower modularity in MB (0.324) than in OL (0.338).

The network indices (BC and CC), along with a large number of nodes (264) and edges (3,918) between taxa, suggest that the *Calditrichaceae*, *Bacteroidetes BD2-2*_Unclassified, *Flavobacteriaceae*, *Sulfurovaceae*, *Anaerolineae*_Unclassified, *γ-Proteobacteria*_Unclassified, *Sedimenticolaceae*, *Pirellulaceae*, *Anaerolineaceae*, *PHOS-HE36*, *MSBL5*_Unclassified, *Milano-WF1B-44*_Unclassified and *Desulfobulbaceae* members play a key role in the benthic environment of MB (Figure 6A). In OL zone, the network consisted of 223 nodes and 3,548 edges. Here, *Thermoanaerobaculaceae*, Bacteria_Unclassified, *Desulfobacteraceae, γ-Proteobacteria*_Unclassified, *Desulfobulbaceae*, *RBG-16-55-12*_Unclassified, *Bacteroidetes BD2-2*_Unclassified, *δ-Proteobacteria*_Unclassified and *Halieaceae* were significantly correlated with each other (Figure 6B).

PICRUST analyses considering KEGG level 3 categories and, a relative abundance greater than >1.0% of the total detected pathways was defined as a dominant pathway. With this criterion, 10 and 4 dominant pathways were stablished in MB and OL, respectively (Supplementary Figure S7). Most of the genes were involved in transporters (average 4.9%), unclassified/poorly characterized (3.6%), ABC transporters (2.9%), DNA repair and recombination proteins (2.4%), signal transduction (2.2%), Purine metabolism (1.9%), bacterial motility proteins (1.9%), Secretion system (1.8%), translation_ribosome (1.8%), and function unknown (1.7%). Among them, transporters, unclassified/poorly characterized, ABC transporter and DNA repair and recombination proteins were remarkably higher in MB (Supplementary Figure S8).

## 4. Discussion

The northern Chilean coast is especially sensitive to the oxygen decline and pollution increase due to both raw sewage input from urban areas and intensive maritime traffic closely linked with mining transport, leading to significant implications on the accumulation of pollutants in marine sediments (Valdés et al., 2014; Castillo et al., 2019). Which in turn could substantially affect microbial communities in the biogeochemical transformation of TMs, leading to modifications to ecosystem processes and functions (Glass et al., 2015; Coclet et al., 2021). To unravel the specific implications for microbial assemblages and their driving forces in the benthic environment, this work goes a step further using 16S rRNA gene sequencing at DNA and RNA (cDNA) molecular levels to comprehensively explore both structure and potential functions as well as provide a first insight into the prokaryotic ecology from two contrasting coastal zones of northern Chile, both of which are characterized by major upwelling or riverine inputs.

### 4.1. Integrated influence of the environmental heterogeneity-driven benthic microbial assemblages

To understand the patterns of microbial assemblages, determining the environmental driving factors is needed, which may play an important role in shaping the community (Tamburini et al., 2020). To the best of our knowledge, there are few studies that have addressed the cumulative effects of oxygen-deficient waters on geochemical metal fractioning in Chilean benthic coastal environments. Thus, this study makes an effort to understand the distributions of Cu and Fe fractions from marine sediments, which is essential in determining the mobility and potential toxicity of TMs.

Though the variation in fraction distributions was observed, recording higher concentrations the MB than in OL, the RF (considered an inert, immobile metal phase) was dominant in both TMs of the sampled zones. Chakraborty et al. (2016) reported that concentrations of Cu associated with F3 and F4 in the Arabian Sea sediments were higher than their concentrations in the F1 and F2. As such, their actual bioavailability is likely to be rather low (chemically stable) and less harmful to the quality of the benthic environment (Cyriac et al., 2021). In addition, the lability of Cu in sediment gradually decreased with decreasing DO levels of the bottom water, which in turn could increase their association with sedimentary OM (Sander et al., 2015). Therefore, we considered that the lower hydrodynamics of MB likely explained the higher levels of TMs when compared to OL, which is physically closer to the discharge of the Loa River.

Besaury et al. (2013) also reported decrease in the ratio of available/total copper concentration alongside an increase of total organic carbon in marine sediments of Chañaral Bay in northern Chile. Thus, it is possible that the low-oxygen levels recorded coupled with the high quantity of OM previously reported both MB and OL zones (Isla et al., 2010; Castillo et al., 2019) increase the geochemical sequestration of TMs and therefore controls bioavailable concentrations due to formation of non-labile complexes in the sediments (Strom et al., 2011; Cyriac et al., 2021). Nevertheless, naturally occurring processes linked to changes in pH, salinity, decomposition and mineralization can favor the release of mobile metal fractions from the sediment to the overlying water, facilitating bioaccumulation processes within the benthic food webs (Chakraborty et al., 2016). In this context, regular assessment to control metal pollution in the coastal environment is recommended (Cyriac et al., 2021). Moreover, marine sediments under high anthropogenic pressure constantly increase HM concentrations, which may lead to significant additional pollution (Jacquiod et al., 2018; Jroundi et al., 2020).

The strategy of using eDNA and eRNA in parallel, revealed the previously unappreciated potential that both active and total microbial communities displayed contrasting patterns and respond differently to environmental heterogeneity gradient. In MB, both Cu and Fe fractions induced significant microbial communities shifts of *Desulfarculales*, *Ignavibacteriales*, *Xanthomonadales*, *Aminicenantales*, *Pseudomonales*, and *Lactobacillales*, whereas *Pirellulales*, *Betaproteobacteriales*, and *Thermoanaerobaculales* were closely related to changes in DO, salinity, and temperature. In contrast, only these two latter factors showed a significant correlation with *Pirellulales* and *Thermoanaerobaculales* in OL, implying that multiple interacting factors in similar environments could have a specific response at the microbial level. This is consistent with previous studies that have demonstrated the clear structuring effect of DO, salinity and temperature in different environments, therefore explaining a significant portion of global microbial community distribution patterns (Zinger et al., 2011; Rodríguez et al., 2018; Hoshino et al., 2020). In the northwestern part of the Gulf of Mexico, the observed pattern was driven primarily by environmental gradients associated with local oceanographic parameters closely linked to water column depth, nutrients, temperature, salinity, OM, DO, and HMs (Jiménez et al., 2018; Rodríguez et al., 2021). Such spatially structured environmental gradients allow micro niches development and promote the coexistence of microbial taxa that are considered regulators of ocean ecosystem services (Fortunato et al., 2012; Cavicchioli et al., 2019).

While environmental gradients clearly affect microbial abundance and activity, much of the spatial variation in microbial activity is linked to pollutant distribution and mobility (Quero et al., 2015; Jacquiod et al., 2018). In this study, Spearman’s rank correlation and RDA analyses, suggested that MB’s microbial structuration seemed to be mainly driven by metal fractioning of Cu and Fe, whereas the major environmental determinants in OL were Fe fractioning, though statistically insignificant (Figure 5; Supplementary Figure S6). It is noteworthy that Coclet et al. (2021) found that Cu, Mn, and Zn modulated the predicted functional profiles of biofilm communities in an anthropized coastal area. These findings further support previous observations of the structural role of metals in other coastal microbial patterns (Jacquiod et al., 2018; Coclet et al., 2019), and even complimented *via* network analysis in different aquatic environments.

Regarding the TMs effects observed in OL, the high water-sediment flow from the Loa River during altiplanic rain could have resulted in an increase in the mass effect and homogenization of sedimentary environmental conditions on the coastal, resulting in decrease in the differential of microbial response. Overall, quantitative evidence supported that the majority of each locale’s (MB and OL) taxa is strongly associated with specific environmental features, suggesting that spatial heterogeneity linked to specific variables of study areas could affect the microbial community assemblage (Zhang et al., 2021). A possible explanation is that the different microbial assemblages have developed distinct mechanisms to adapt to environmental heterogeneity of study zones, driving stabilization of the functional structure and maintenance of ecological services (Zinger et al., 2011; Cavicchioli et al., 2019; Hoshino et al., 2020).

Indeed, the major functional pathways at both zones were involved in transporters, BC transporters, DNA repair, and recombination proteins, and signal transduction, which were previously found in an aquatic system affected by acid mine drainage. Such a finding highlights that transporters are involved in metal tolerance and detoxification (Coclet et al., 2021), particularly multiple efflux systems that allow microbes to sense and respond to variable environmental conditions and adapt to the environment (Chen et al., 2015). These results are fundamental value because understanding how different local regimes of environmental variability modify the spatial structure of benthic communities may provide a starting point to consider how communities could respond to future climate changes and metal pollution scenarios in reduced oxygen environments (Cavicchioli et al., 2019).

However, these results must be interpreted carefully because it should be recognized that, while microbial community structure could be influenced by variables measured within this study, additional variables that were not measured (e.g., nitrogen, sulfur content) could also affect microbial community structure (Farías et al., 2004; Hoshino et al., 2020). Therefore, future work should aim to employ an integrated metatranscriptomic and metagenomic approach to functional inferences. The combination of these methods can concurrently investigate the transcriptional patterns of specific microbes, revealing their overall functional and metabolic activities within the whole community (Stewart et al., 2012).

### 4.2. Spatial co-occurrence patterns of benthic microbial assemblages

The benthic microbial assemblages from two contrasting coastal study zones revealed strong spatial variation patterns with a large fluctuation in microbial communities in terms of taxonomic composition. This contributed to the keystone taxa groups that formed, distinguishing two strong module hubs from each study area. The taxa that are defined as module hubs and connectors of the network are regarded as keystone taxa due to their role in maintaining network structures, and they may play a role in maintaining ecosystem stability (Cui et al., 2019).

Overall, the keystone taxa we observed in the benthic microbial community may expand the range of possible metabolic roles into sediments, elucidating their ecological niches. A clear example of this was the identification of *Calditrichaceae* (phylum *Calditrichaeota*) in the sediments of MB, which probably pointed to a stronger line of evidence for the importance of this family in marine sediments, as it has been previously reported as an unrecognized but significant player in geochemical element cycling that is able to degrade detrital proteins (Marshall et al., 2017). Many members of the family *Flavobacteriaceae* have been isolated from marine environments and also have the capacity to degrade high-molecular-weight organic polymers, including cellulose, chitosan, and proteins (Yang et al., 2020). Moreover, this particular family has the ability to tolerate toxic effects of certain metals (Quero et al., 2015; Tamburini et al., 2020).

The reconstruction of metagenome-assembled genomes in the microbial-mediated sulfur cycle determined that *Sedimenticolaceae* possess near-complete, encoded pathways for denitrification (Vavourakis et al., 2019). Indeed, along with *Desulfobulbaceae*, *Sedimenticolaceae* was the most abundant family reported in association with marine sediments from Chilean salmonid farms (Miranda et al., 2020). Furthermore, some of these families including *Desulfobacteraceae* were observed in the permanent anoxic/hypoxic sediment in the deepest part of the Baltic Sea, which highlighted active functional traits related to OM degradation, reduction of sulfate, and methanogenesis (Broman et al., 2017).

Network analysis also facilitated further identification of *Thermoanaerobaculaceae* as the major representative of OL, which have been previously described as thermophilic in the sediments of Boka Kotorska Bay (Montenegro, Croatia; Jokanović et al., 2021). Interestingly, some prominent keystone taxa in our study belonged to the bacteria rare subcommunity, which indicated that these microbes may compose the hidden backbone of microbial communities. Furthermore, the beta-diversity analysis showed a clear distinction between the microbial assemblages in MB and OL with significantly higher richness (Chao1; *p* < 0.05) in the DNA samples compared to the RNA samples across sampled water-depths.

In accordance with discriminant analysis for the spatial variation between the sampled zones revealed that *Beggiatoaceae*, *Carnobacteriaceae*, *Tenderiaceae*, *Saprospiraceae*, *Family XI_2I* and *Nitrosococcaceae* were representative of OL. For MB, *Enterobacteriaceae*, *Corynebacteriaceae*, *Latescibacteraceae*, *Clostridiales Incertae Sedis*, *Iamiaceae*, *Clostridiaceae* and *Solirubrobacterales_67-14* were the families responsible for the observed changes, indicating the dispersal of fecal bacteria from the anthropogenically affected coastal area and therefore representing a reservoir of pathogenicity potentially harmful to human health (Nogales et al., 2011). Indeed, feco–oral route is the most important route of transmission especially for resistant pathogens of the family *Enterobacteriaceae* (Aslam et al., 2018).

Based on several active bacteria detected, such as *Burkholderia*, *Campylobacter*, *Clostridium*, *Enterobacter* and *Pseudomonas* groups, which are resistant to various antibiotics, the influence of MB environment is also of concern Mainly due to pose a serious threat to global human, animal, and environmental health, a concern recognized by numerous important organizations such as the World Health Organization (Aslam et al., 2018; Miranda et al., 2018). However, the above differential assemblage compositions among study zones also suggest the cooperation between all organisms, potentially translating into success of the whole ecosystem (Cardoso et al., 2017), because they reveal the performed functions that are either similar or complementary to each other and may co-occur due to shared and preferred environmental conditions (Jroundi et al., 2020).

Previous studies that have analyzed eRNA in parallel with eDNA also reported significantly higher values in the overall community. What is more, DNA sampling (living, dead, eaten, and recently moved through the sediments) is logistically simpler than sampling RNA or the active community, which usually contains fewer communities (review Birrer et al., 2018; Pawlowski et al., 2022 for more details); it should be noted that this is not always the case because eRNA is usually more sensitive, particularly for biosecurity applications, where detecting viable non-indigenous organisms is critical. Therefore, highlighting new opportunities for integrating functional genomics into environmental monitoring programs that may inform management actions in order to represent real-time ecological change and impacts could serve as a predictive tool for identifying at-risk habitats (Bowers et al., 2021), especially as sequencing costs decline. Therefore, the findings of this study help to lay the groundwork for scoping eRNA in parallel with eDNA applied to significantly enhance the spatial and functional understanding of real-time microbial assemblages and, in turn, to increase the acuity of biomonitoring programs key to responding to immediate management needs for the marine environment.

Campbell and Kirchman (2012) explain the decoupling between abundance and microbial activity as a result of grazing and virus lysis. Interestingly, sediment chemistry can affect the efficiency of nucleic acid extraction and PCR amplification, especially in polluted sediments like those of MB (Valdés et al., 2005; Valdés, 2012; Castillo et al., 2019), where organic and inorganic compounds bind to the sediment (Sander et al., 2015). It is also important to note that the potential bias in the differential nucleic acids extraction and amplification could generate an underestimation of some groups diversity (Cardoso et al., 2017; Birrer et al., 2018). For this reason, the libraries were normalized by an equal number of sequences before performing calculations or statistical tests.

When exploring the spatial co-occurrence patterns of microbial assemblages between study zones, two distinct module hubs (regions densely connected) were clearly observed in both MB and OL networks, indicating that habitat significantly influenced the microbial co-occurrence networks (Cui et al., 2019; Felipe-Lucia et al., 2020). In fact, the habitat preference of microorganisms may play a vital role in determining their co-occurrence patterns (Chaffron et al., 2010; Jroundi et al., 2020). It was found that the microbial network in the OL benthic zone showed higher complexity (given the value of modularity) compared to the MB (as described in the results). The lower modularity network in MB, could mean that its communities are more vulnerable and sensitive to various disturbances, demonstrating that, in high-stress environments, their communities could break down (Banerjee et al., 2018; Philippot et al., 2021).

That said, the high modularity in OL mainly indicates diverse micro-niches and structured microbial communities (Zhang et al., 2021), characteristic of large complex systems that imply a higher ecological degree of stability under perturbations. This improves efficiency in transferring information, energy, and resources of microbial communities, regard to sediment nutrient conversion and pollutant biodegradation (Santolini and Barabási, 2018; Philippot et al., 2021). One explanation for the high spatial connectivity and the selection of the microbe pool in the coastal area surrounding the OL may also be attributed to the Loa River, which acts as a true green/life corridor that runs across the hyper-arid core of the Atacama Desert also known as the dry limit for life on Earth, helping to establish specific networks (Zárate et al., 2020). In this way, the input may support a distinct set of microbial processes that remain unstudied (Fortunato et al., 2012; Fazi et al., 2020; Jroundi et al., 2020).

For instance, in the coastal wetlands of both the Yellow River Delta (China) and the northern Adriatic Sea (Italy), it has been emphasized that river input altered microbial structure patterns (Fazi et al., 2020; Zhao et al., 2020). Thus, the results of this study are consistent with the environmental microbiomes that highlight associations between prokaryotic consortia, representing potential metabolic interactions with habitat transition (Chaffron et al., 2010; Petro et al., 2017) and local heterogeneity that is usually characterized by highly productive systems. In addition, in the Yap Trench (Western Pacific Ocean), the formation of multiple modules that respond to the environmental heterogeneity in sediments was also reported, providing evidence for ecological niche differentiation (Zhang et al., 2021). This study also highlights the relevance of the 66.2% of the resident microbial communities that were shared in the study areas, suggesting that they harbor a persistent functional pool of ecological potential that may be temporally consistent and regionally transferable (Clark et al., 2020). Therefore, it is possible to propose that these common taxa could be more useful for the development of coastal health assessment and biological monitoring tools (Sun et al., 2013; Quero et al., 2015). It is crucial to clarify the processes underlying changes microbial communities and ecosystems in response to environmental perturbations (Nogales et al., 2011; Cavicchioli et al., 2019).

## 5. Conclusion

This study demonstrated that microbial assemblages from two contrasting benthic coastal zones in northern Chile were highly diverse and strongly regulated by a combination of different environmental factors including Cu, Fe, dissolved oxygen, salinity, and temperature, Such conditions led to heterogeneous distribution with rare communities that contributed to keystone taxa in networks similar to large complex systems, which potentially serve a key role in ecosystem function, though the temporal dynamics of which requires further description and understanding. Additionally, we highlight the possibility that the keystone taxa identified could serve as a basis for future monitoring programs of contaminated environments. This is especially true for the microbial processes occurring in the sediment of Mejillones Bay, whose bacteria could play an important role in the region exchange of antibiotic resistance. Therefore, the eRNA in parallel with eDNA approach utilized here has the capacity to significantly enhance the spatial and functional understanding of real-time microbial assemblages and, in turn, would have the potential to increase the acuity of biomonitoring programs that are key to addressing the global concern of antimicrobial resistance from the coastal environment. Hence, future work should aim to further experimental validation and cultivation-based approaches will be required to identify consistent responses of such indicator taxa, employing an integrated metagenomic and metatranscriptomic approach to reveal functional and metabolic activities within the whole microbial benthic community.

## Data availability statement

The datasets presented in this study can be found in online repositories. The names of the repository/repositories and accession number(s) can be found in the article/Supplementary material.

## Author contributions

AZ, CD, and JV proposed and designed the study. AZ organized and performed field sampling. SV and AZ analyzed the samples. GI and JU performed the bioinformatic analyses. VM, AC, and AZ analyzed and interpreted the results and participated in manuscript writing. All authors contributed to the article and approved the submitted version.

## Funding

This work was supported by a Scientific and Technological Research Scholarship for Doctoral Studies (CONICYT-PCHA/Doctorado Nacional/2015–21150407) and Centro de Bioingeniería y Biotecnología (CeBiB) FB0001. The funders had no role in study design, data collection and analysis, decision to publish, or preparation of the manuscript.

## Conflict of interest

The authors declare that the research was conducted in the absence of any commercial or financial relationships that could be construed as a potential conflict of interest.

## Publisher’s note

All claims expressed in this article are solely those of the authors and do not necessarily represent those of their affiliated organizations, or those of the publisher, the editors and the reviewers. Any product that may be evaluated in this article, or claim that may be made by its manufacturer, is not guaranteed or endorsed by the publisher.
