# Supplementary material for: Spatial co-occurrence patterns of benthic microbial assemblage in response to trace metals in the Atacama Desert Coastline
Source: Front Microbiol. 2023 Jan 16;13:1020491. doi: 10.3389/fmicb.2022.1020491 (PMC9885135; doi:10.3389/fmicb.2022.1020491)
Supplement: Supplementary file 1 [file Data_Sheet_1.PDF]

Supplementary Material

Supplementary Figures and Tables

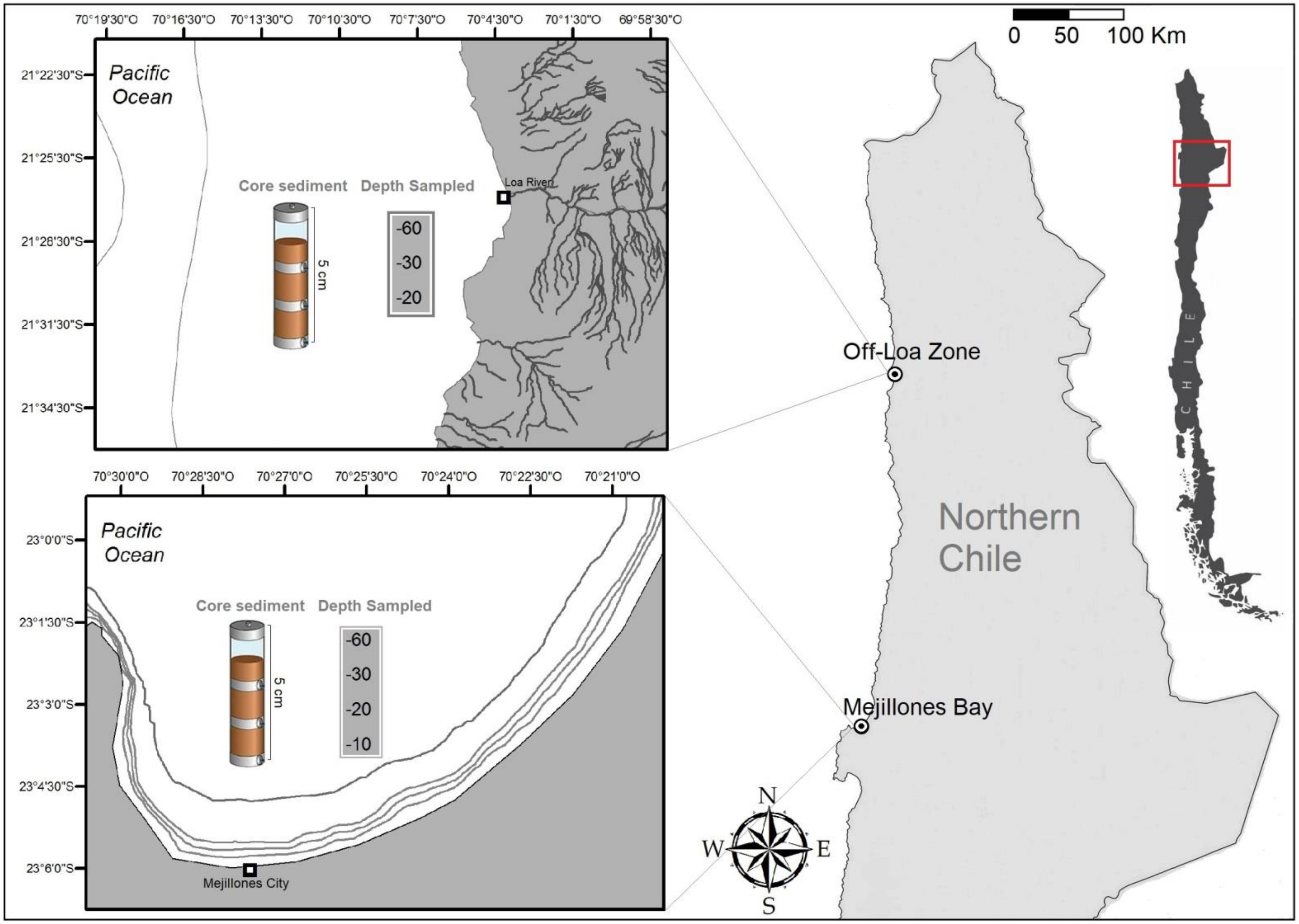

Figure S1. Sampling sites in northern Chile. Map showing the location of sampling sites in coastal Mejillones Bay and Off-Loa River.

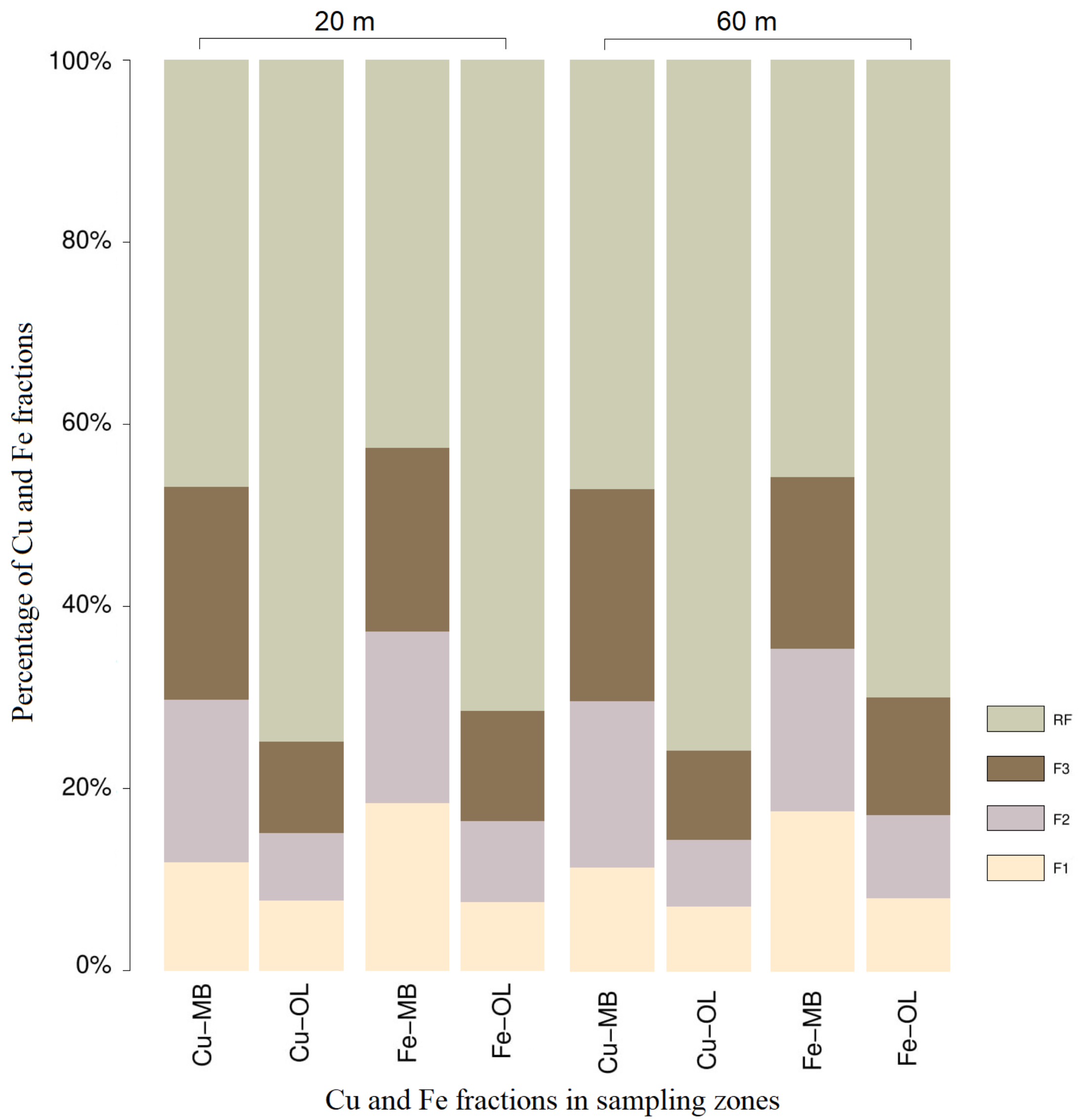

Figure S2. Spatial distribution of the extracted Fe and Cu fractions from Mejillones Bay (MB) and Off-Loa River zone (OL) across the water depth sampled.

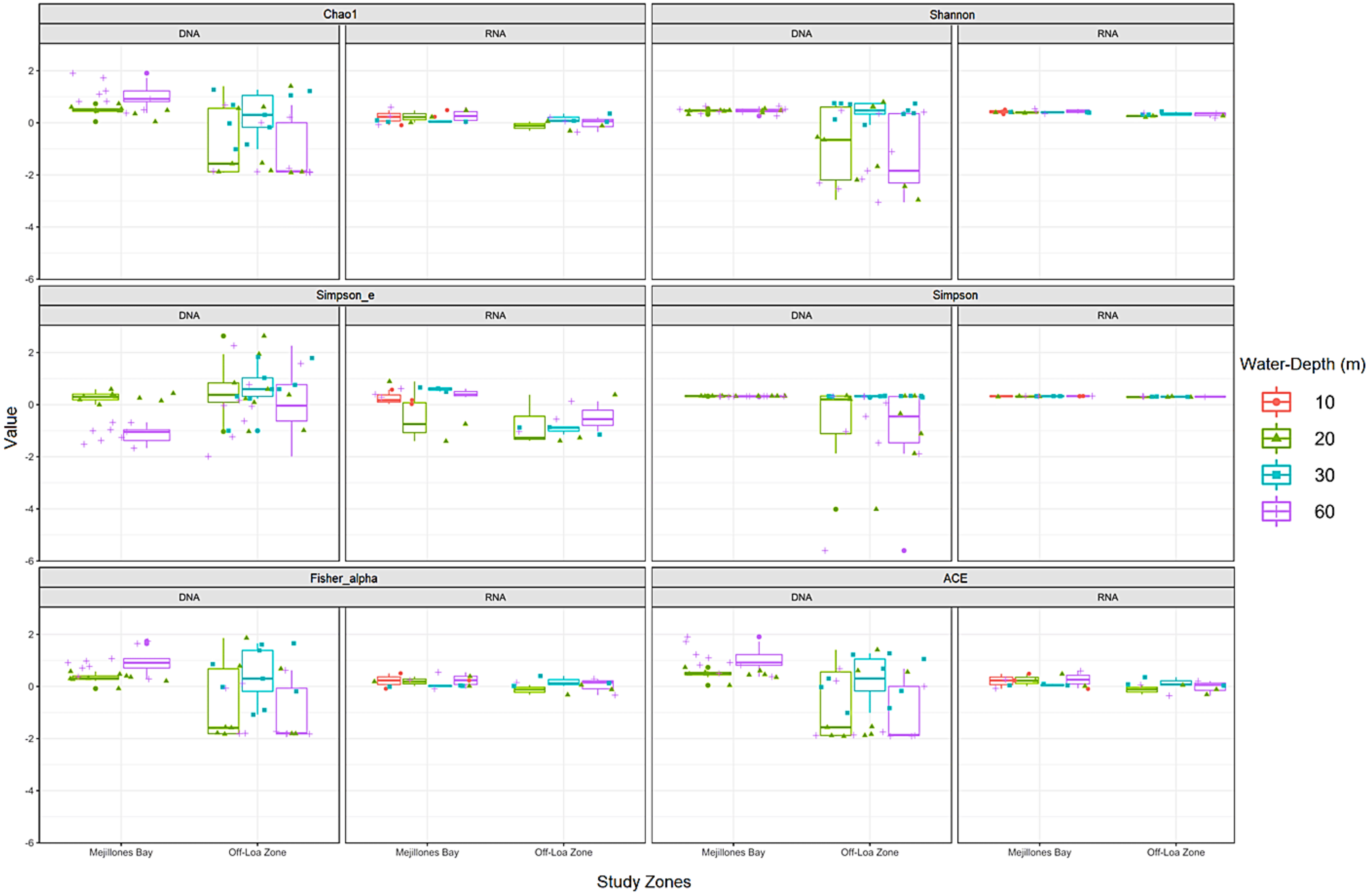

Figure S3. Boxplot showing the difference in diversity indices (ACE, Simpson, Shannon, and Chao1 recorded p < 0.001, Kruskal-Wallis test) among eDNA and eRNA from Mejillones Bay and Off-Loa River zone across the water depth gradients.

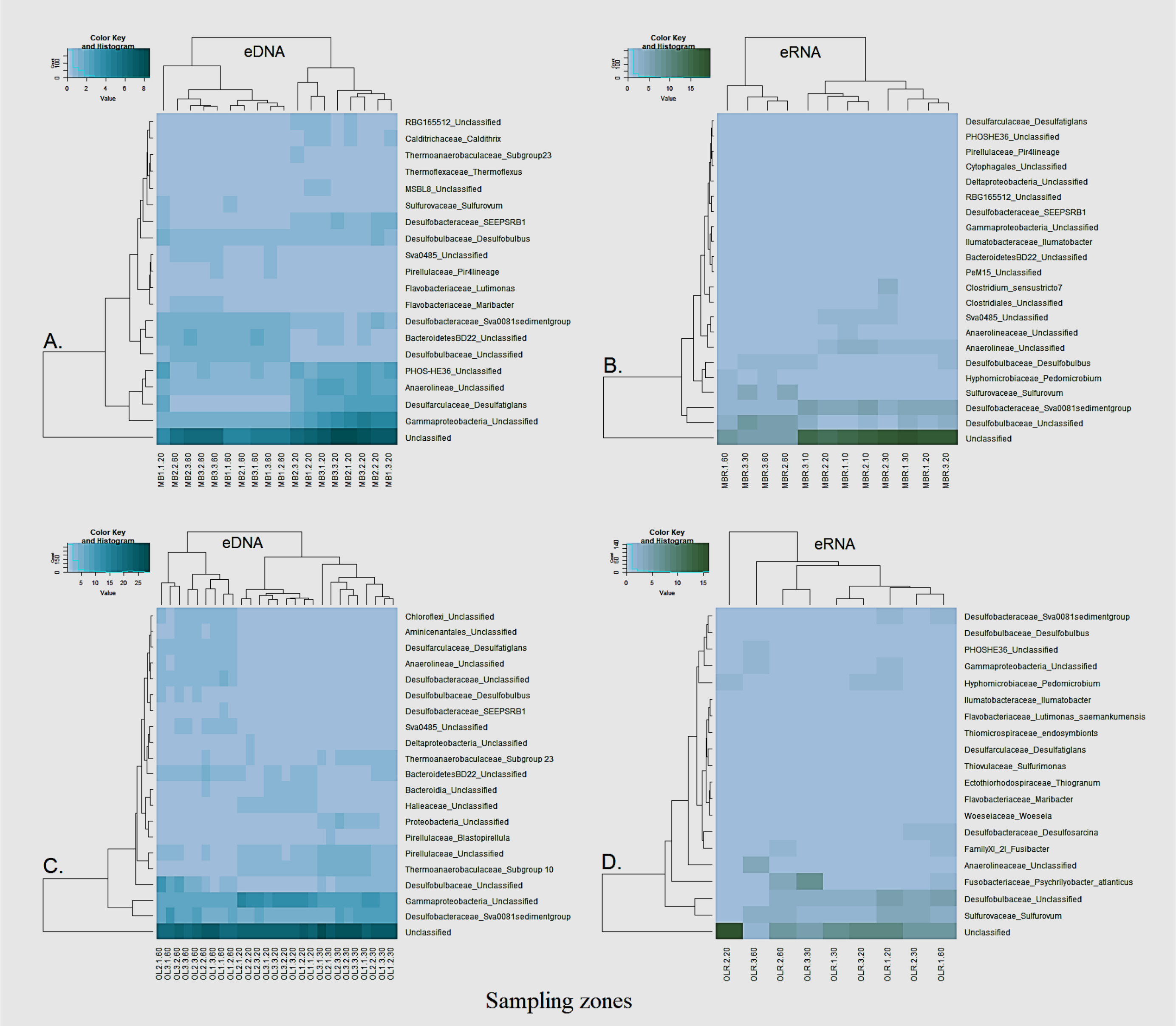

Figure S4. Heatmap indicating the microbial communities from eDNA (A, C) and eRNA (B, D) with a mean relative abundance of ≥1% unclassified taxa from the genus to the species level in the Mejillones Bay (top) and Off-Loa River zone (bottom).

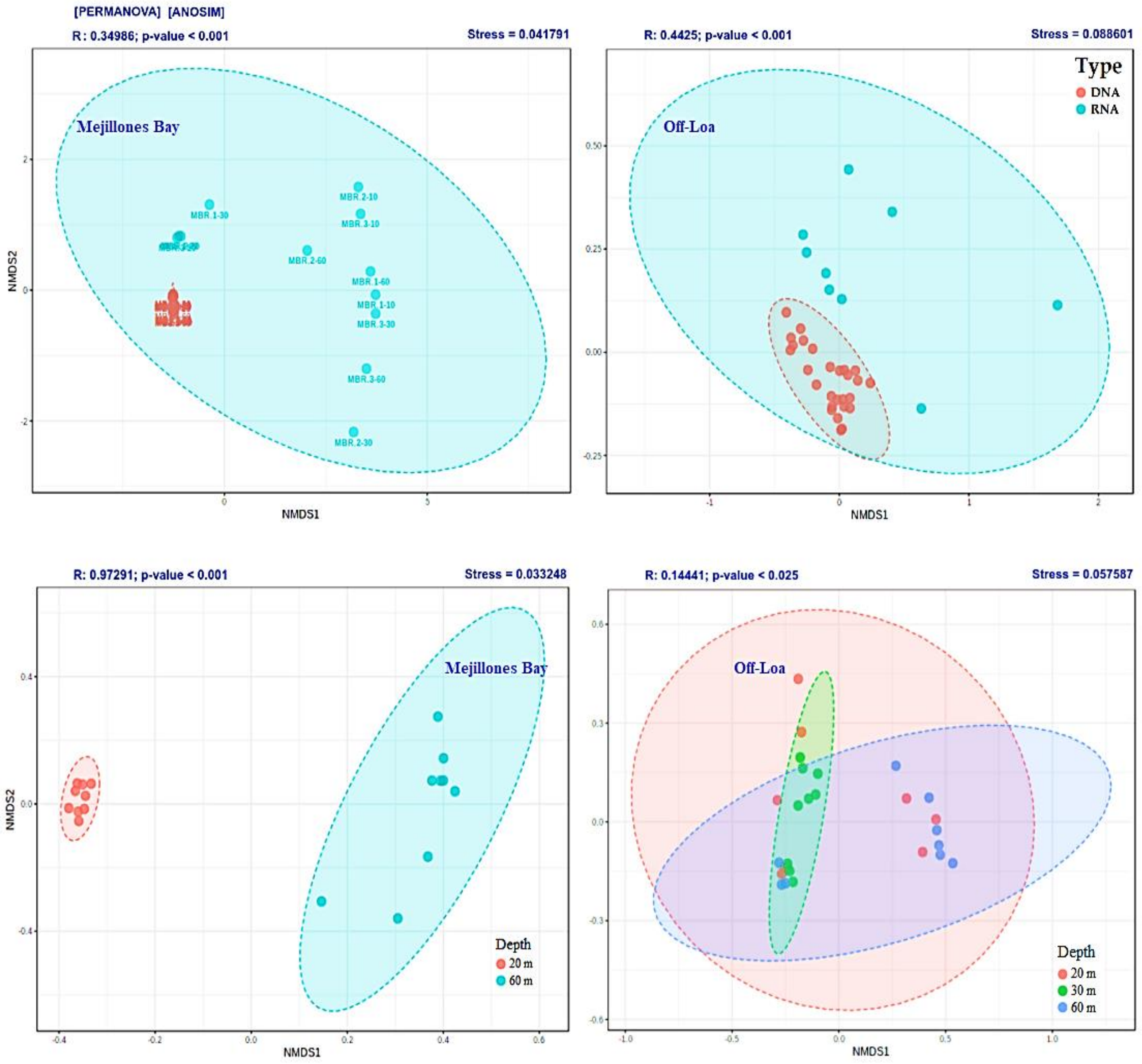

Figure S5. Non-metric multidimensional scaling (NMDS) ordination about the differences in present (eDNA) and active (eRNA) prokaryotic community in sediment samples of Mejillones Bay and (right) Off-Loa River zone (left) across the water-depth.

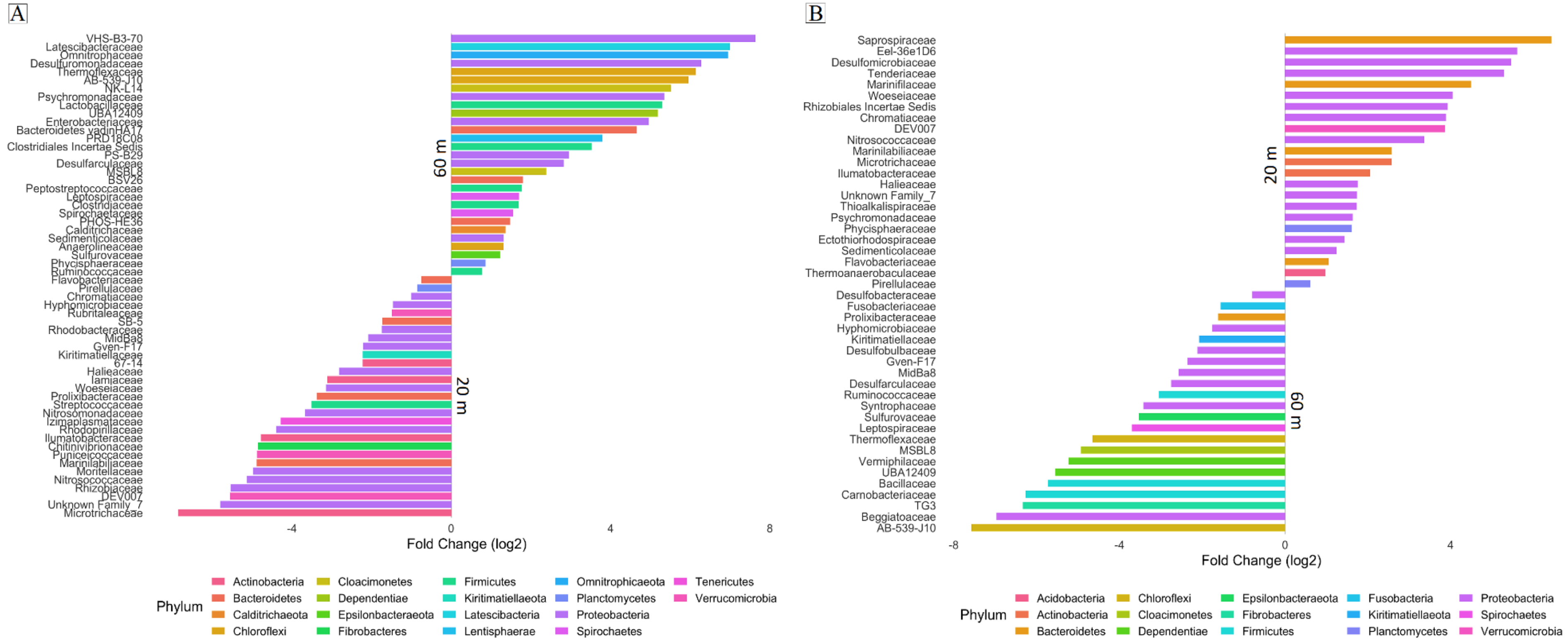

Figure S6. DESeq2 analysis showing the family differential abundance based on depth in (A) Mejillones Bay and (B) Off-Loa River zone.

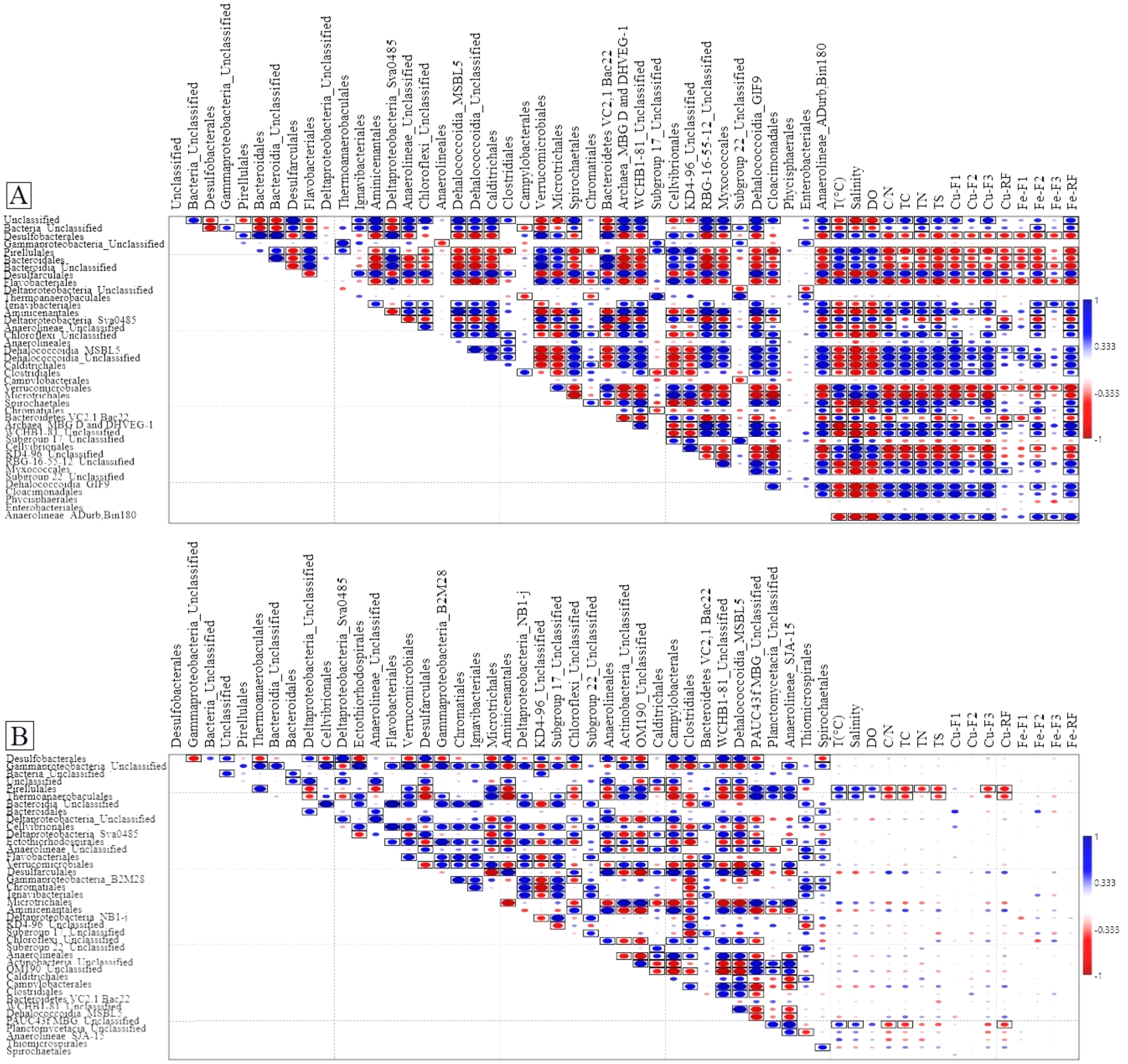

Figure S7. Spearman's correlation diagram between the dominant benthic microbial family and environmental variables in (A) Mejillones Bay and (B) Off-Loa River zone. The colors in the panel represent the correlation coefficient, increasing from red (negative) to blue (positive). Framed boxes refer to significant correlations ($p < 0.05$). Bubble size indicates the regression coefficient, increasing from small to large.

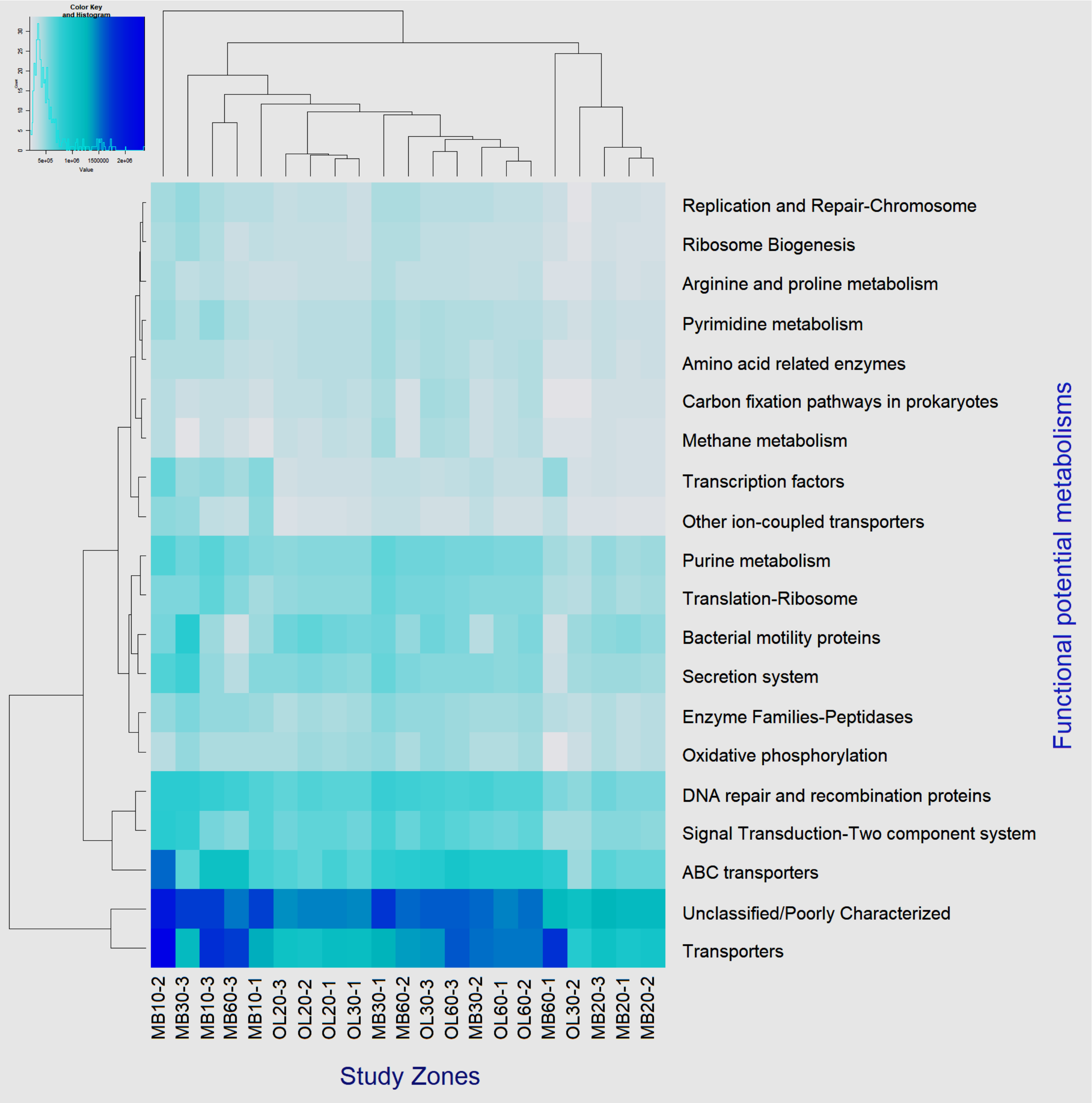

Figure S8. Heatmap based on the relative abundance (> 0.5%) and distribution of functional metabolic genes in the benthic microbial community at two sampling zones.

Table S1. Taxonomic annotation levels

Sample ID	Level	Classified (%)	Unclassified (%)
MB	Kingdom	82.10	17.90
	Phylum	75.17	24.83
	Class	72.67	27.33
	Order	55.42	44.58
	Family	43.29	56.71
	Genus	25.48	74.52
	Species	0.69	99.31
MBR	Kingdom	76.51	23.49
	Phylum	72.17	27.83
	Class	69.76	30.24
	Order	54.04	45.96
	Family	43.45	56.55
	Genus	27.82	72.18
	Species	0.90	99.10
OL	Kingdom	81.42	18.58
	Phylum	76.93	23.07
	Class	72.67	27.33
	Order	53.45	46.55
	Family	42.99	57.01
	Genus	27.33	72.67
	Species	0.72	99.28
OLR	Kingdom	81.92	18.08
	Phylum	78.37	21.63
	Class	76.83	23.17
	Order	62.40	37.60
	Family	51.66	48.34
	Genus	34.41	65.59
	Species	2.32	97.68

Table S2. Network features and taxonomy of "keystone taxa" in the Mejillones Bay and Off-Loa River zone.

Study Zone	ASVs Level Family	Network features				Taxonomy			
		Betweenness Centrality	Closeness Centrality	Degree	Modularity Class	Class	Order	Genus	% Relative Abundance
Mejillones Bay	*Calditrichaceae*	799.7	0.629	41	5.0	*Calditrichia*	*Calditrichales*	*Calorithrix*	0.83
	Bacteroidetes_BD2-2	601.0	0.691	75	5.0	*Bacteroidia*	*Bacteroidales*	Unclassified	3.25
	Flavobacteriaceae	572.1	0.783	85	5.0	*Bacteroidia*	*Flavobacteriales*	Unclassified	1.88
	Sulfurovaceae	566.0	0.491	28	2.0	*Campylobacteria*	*Campylobacterales*	*Sulfurovum*	0.67
	Anaerolineaceae	476.0	0.474	49	2.0	*Anaerolineae*	*Anaerolineales*	Unclassified	1.16
	γ-Proteobacteria	410.2	0.523	29	5.0	*γ-Proteobacteria*	Unclassified	Unclassified	6.70
	Sedimenticolaceae	349.3	0.588	73	5.0	*γ-Proteobacteria*	*Chromatiales*	*Sedimenticola*	0.52
	Pirellulaceae	345.9	0.607	67	5.0	*Planctomycetacia*	*Pirellulales*	Unclassified	6.12
	PHOS-HE36	323.0	0.557	29	4.0	*Ignavibacteria*	*Ignavibacteriales*	Unclassified	1.22
	MSBL5	312.1	0.381	36	2.0	*Dehalococcoidia*	*MSBL5*	Unclassified	0.95
	Milano-WF1B-44	307.5	0.666	82	5.0	*γ-Proteobacteria*	*Milano-WF1B-44*	Unclassified	0.18
	Desulfobulbaceae	237.8	0.472	27	2.0	*δ-Proteobacteria*	*Desulfobacterales*	*Desulfobulbus*	3.58
Off-Loa Zone	*Thermoanaerobaculaceae*	598.0	0.574	48	2.0	*Thermoanaerobaculia*	*Thermoanaerobaculales*	*Subgroup_23*	4.69
	Bacteria_Unclassified	297.4	0.543	62	2.0	Unclassified	Unclassified	Unclassified	13.30
	Desulfobacteraceae	290.4	0.733	17	1.0	*δ-Proteobacteria*	*Desulfobacterales*	Unclassified	11.82
	γ-Proteobacteria	201.8	0.558	64	2.0	*γ-Proteobacteria*	Unclassified	Unclassified	14.05
	Desulfobulbaceae	175.2	0.592	64	2.0	*δ-Proteobacteria*	*Desulfobacterales*	Unclassified	4.01
	RGB-16-55-12	158.8	1.000	49	1.0	Unclassified	Unclassified	Unclassified	0.16
	Bacteroidetes_BD2-2	155.5	0.404	11	4.0	*Bacteroidia*	*Bacteroidales*	Unclassified	1.74
	δ-Proteobacteria	111.3	0.621	29	3.0	Unclassified	Unclassified	Unclassified	2.02
	Halieaceae	105.3	0.500	50	3.0	*γ-Proteobacteria*	*Cellvibrionales*	Unclassified	1.79

Table S3. Environmental variables to Mejillones Bay and Off-Loa River zone coastal marine locations across the water-depth gradients.

Zone		Sample ID	Depth (m)		Salinity (PSU)	DO (ml/l)	T (°C)	TC (%)	TN (%)	TS (%)
Sampling coastal marine zone in northern Chile	Mejillones Bay	MB1-10		Mean	34.841	1.155	15.19	NA	NA	NA
		MB2-10	10	SD	0.037	0.104	0.11	NA	NA	NA
		MB3-30		Max	34.876	1.255	15.31	NA	NA	NA
		MB1-20		Mean	34.862	1.110	15.03	5.25	2.92	0.83
		MB2-20	20	SD	0.010	0.041	0.02	5.11	2.77	0.77
		MB3-20		Max	34.870	1.146	15.05	5.10	2.88	1.04
		MB1-30		Mean	34.868	1.545	15.23	NA	NA	NA
		MB2-30	30	SD	0.011	0.042	0.02	NA	NA	NA
		MB3-30		Max	34.879	1.577	15.26	NA	NA	NA
		MB1-60		Mean	34.840	0.160	14.07	5.25	3.43	1.13
		MB2-60	60	SD	0.001	0.003	0.00	5.45	3.80	1.22
		MB3-60		Max	34.841	0.163	14.07	5.61	3.82	1.19
	Off-Loa Zone	OL1-20		Mean	34.830	0.078	12.78	4.82	0.26	1.11
		OL2-20	20	SD	0.004	0.014	0.02	5.02	0.35	0.98
		OL3-20		Max	34.840	0.088	12.80	4.97	0.35	1.24
		OL1-30		Mean	34.830	0.073	12.77	NA	NA	NA
		OL2-30	30	SD	0.001	0.007	0.01	NA	NA	NA
		OL3-30		Max	34.830	0.080	12.78	NA	NA	NA
		OL1-60		Mean	34.830	0.065	12.53	4.92	0.44	1.24
		OL2-60	60	SD	0.008	0.005	0.01	5.03	0.45	1.30
		OL3-60		Max	34.840	0.070	12.54	5.08	0.46	1.25

All measurements are made with a Seabird CTD probe 19 Plus. DO values correspond to bottom water concentrations. NA is Not Available.

Table S4. Summary of sediment sequencing results from eDNA and eRNA across the water -depths sampled in the Mejillones Bay and Off-Loa coastal zones.

Study zone	SampleID	Type	Depth (m)	InputReads	PostFilter	DenoisedF
Mejillones Bay	MB1.1	DNA	20	70997	69740	48784
	MB1.2			71611	70338	49498
	MB1.3			67736	66638	46794
	MB2.1			75340	73987	52937
	MB2.2			68830	67720	49077
	MB2.3			62258	61195	42624
	MB3.1			74498	73224	52672
	MB3.2			70113	68983	47631
	MB3.3			72547	71284	51912
	MB1.1		60	71038	69840	42949
	MB1.2			81476	80068	50418
	MB1.3			73827	72567	48372
	MB2.1			69955	68804	44684
	MB2.2			68820	67695	43421
	MB2.3			75241	73968	53740
	MB3.1			84946	83538	67893
	MB3.2			79233	77875	58906
	MB3.3			71454	70244	58298
	MBR.1	RNA	10	64853	52329	36307
	MBR.2			58556	48288	34167
	MBR.3			57000	44538	29523
	MBR.1		20	71606	58846	46196
	MBR.2			57594	46566	30988
	MBR.3			60489	49650	37043
	MBR.1		30	52869	43682	32737
	MBR.2			61089	50108	36945
	MBR.3			61407	49129	35908
	MBR.1		60	58996	49255	35603
	MBR.2			53455	43677	31522
	MBR.3			69127	57342	42350
Off-Loa	OL1.1	DNA	20	35000	32123	32076
	OL1.2			35000	32047	29590
	OL1.3			35000	32113	31878
	OL2.1			35000	33323	33153
	OL2.2			35000	31840	31578
	OL2.3			35000	31477	31188
	OL3.1			35000	31498	31322
	OL3.2			35000	31902	30970
	OL3.3			35000	31554	29281
	OL1.1		30	35000	31787	30408
	OL1.2			35000	31423	31033
	OL1.3			53309	43555	30750
	OL2.1			35000	30804	29066

	OL2.2			35000	32173	30507
	OL2.3			58086	48183	35074
	OL3.1			34915	31896	30901
	OL3.2			35000	32017	29877
	OL3.3			53675	42595	30246
	OL1.1			35000	31945	31791
	OL1.2			35000	31447	31371
	OL1.3			61172	48827	44830
	OL2.1			35000	31859	31591
	OL2.2		60	35000	31526	31384
	OL2.3			56866	45992	37535
	OL3.1			35000	31737	31591
	OL3.2			35000	32074	31902
	OL3.3			65696	54006	43021
	OLR.1			43139	34368	31028
	OLR.2		20	43331	35373	28581
	OLR.3			43198	35639	32029
	OLR.1			46185	37454	33056
	OLR.2	RNA	30	44583	36395	33021
	OLR.3			41144	32884	29932
	OLR.1			43708	34773	29037
	OLR.2		60	41918	32953	25173
	OLR.3			44270	36030	26285
